# Hierarchical Cobalt Hydroxide and B/N Co-Doped Graphene Nanohybrids Derived from Metal-Organic Frameworks for High Energy Density Asymmetric Supercapacitors

**DOI:** 10.1038/srep43084

**Published:** 2017-02-27

**Authors:** Hassina Tabassum, Asif Mahmood, Qingfei Wang, Wei Xia, Zibin Liang, Bin Qiu, Ruo zhao, Ruqiang Zou

**Affiliations:** 1 Beijing Key Laboratory for Theory and Technology of Advanced Battery Materials, Department of Materials Science and Engineering, College of Engineering, Peking University, Beijing 100871, China

## Abstract

To cater for the demands of electrochemical energy storage system, the development of cost effective, durable and highly efficient electrode materials is desired. Here, a novel electrode material based on redox active β-Co(OH)_2_ and B, N co-doped graphene nanohybrid is presented for electrochemical supercapacitor by employing a facile metal-organic frameworks (MOFs) route through pyrolysis and hydrothermal treatment. The Co(OH)_2_ could be firmly stabilized by dual protection of N-doped carbon polyhedron (CP) and B/N co-doped graphene (BCN) nanosheets. Interestingly, the porous carbon and BCN nanosheets greatly improve the charge storage, wettability, and redox activity of electrodes. Thus the hybrid delivers specific capacitance of 1263 F g^−1^ at a current density of 1A g^−1^ with 90% capacitance retention over 5000 cycles. Furthermore, the new aqueous asymmetric supercapacitor (ASC) was also designed by using Co(OH)_2_@CP@BCN nanohybrid and BCN nanosheets as positive and negative electrodes respectively, which leads to high energy density of 20.25 Whkg^−1^. This device also exhibits excellent rate capability with energy density of 15.55 Whkg^−1^ at power density of 9331 Wkg^−1^ coupled long termed stability up to 6000 cycles.

Supercapacitors or electrochemical capacitors (ECs) have attracted great attention as an emerging class of energy storage devices in recent years due to their high power density and long life span^1^,^2^,^3^. ECs are grouped into two categories of electrical double layer capacitors (EDLCs) and Faradaic capacitors on the basis of charge storage mechanism^4^,^5^. Particularly, EDLCs store charges by the formation of electrical double layers on the surface of electrode materials and their capacitance is mainly contributed by the active surface area, porosity of the carbonaceous electrode materials which possess high power density greater than ~10000 Wkg^−1 ^6,7,8,9,10. However, such electrode materials have limited energy density which hinders their large scale application. In comparison, Faradaic capacitors based on transition metal oxides/hydroxides store charges by reversible redox reactions^1^,^11^, usually exhibiting high capacitance and energy densities with poor cyclic stability due to poor electrical conductivity, low surface area, and extensive aggregation upon cycling^12^,^13^,^14^. Therefore, several attempts are devoted to improve the electrochemical properties by combining carbonaceous conducting materials with nano-sized metal oxides/hydroxides for enhanced capacitor performance^2^. Among many transition metal oxides and hydroxides, Co_3_O_4_ or Co(OH)_2_ nanomaterials are excellent candidates for the EC applications. Up to now, numerous methods have been dedicated to develop nanohybrids for aqueous asymmetric supercapacitors (ASCs), such as Co(OH)_x_CO_3_//CF−CNT^15^ and Co_3_O_4_//NC hybrids etc^16^. Although the performance of these hybrids exhibit great potential but their poor cyclic stabilities hinder the practical applications. The mainly reason lies in the poor interaction at molecular level of two compositions, resulting low stability of metal oxide or hydroxide nanoparticles (NPs) during charge/discharge process between high surface area carbon-based conducting materials and metal oxides/hydroxide NPs^12^,^17^,^18^. To tackle this issue, it is required to enhance the intermolecular interactions and improve the synergistic effects among two key compositions. Recently, a great interest has been drawn to produce the metal-organic frameworks (MOFs) based electrode materials to enhance the capacitance and cyclic life because of their higher surface area, inherently present metallic source, ordered structure and heteroatom in organic ligand^19^,^20^,^21^. Although these MOF-based materials with high surface area and hierarchical porous structures could *in-situ* confine the Faradaic-active metal or metal oxide nanostructures, their electrical conductivity is still far from the expected range for ECs due to their low graphitization^22^,^23^,^24^,^25^,^26^,^27^.

Herein, we concept a zeolitic-imidazolate framework (ZIF) route towards *in-situ* fabrication of Co(OH)_2_ within carbon polyhedron (CP) and B/N co-doped graphene (BCN) nanosheets for this effort. ZIF is a family of MOF materials with imidazolate as ligands, which has been recently employed as a precursor for preparing N-doped carbon with or without metal NPs in the pores^4^,^28^,^29^. Under properly controlled calcination condition, high surface area carbonaceous electrode materials with large number of active sites for electrochemical reactions could be obtained. However, the use of pure ZIF based materials exhibit lower capacitive performance due to poor conductivity as well as inactive metal species. In this regard, the two-dimensional (2D) layered B/N co-doped graphitic (BCN) materials with high conductivity can be employed to enhance the electrochemical properties of ZIF derived materials^30^,^31^,^32^. The segregated co-doping of B and N moieties in carbon can present electron deficiency and electron rich sites, respectively, which can reduce the charge transfer resistance, present different binding sites for improved wettability and enhance the electrical contact between the overlapping sheets as well as enhanced charge storage capability^30^,^31^. In addition, the co-doped graphitic nanosheets with large number of active B and N doped sites along with high surface area could provide active interface in ECs with remarkable capacitance as reported *ca.* 321 Fg^−1 ^33,34,35,36. The key for this target idea is to strongly bond the Faradaic-active metal oxides/hydroxides with BCN nanosheets together. Therefore, we employed *in-situ* method to fabricate β-Co(OH)_2_ based nanohybrids from ZIF-67^19^ nanocrystals and BCN precursors by solution chemistry and high-temperature pyrolysis as illustrated in Fig. 1. The ZIF-67 was particularly selected to tailor β-Co(OH)_2_ to confine redox-active species in highly porous three dimensional (3D) carbon framework to obtain large channels for mass transport as well as higher surface area to increase electrode/electrolyte interface. The β phase of Co(OH)_2_ was synthesized due to its higher capacitance and longtime stability in strong alkaline solution^37^. This ideal composite structure displays perfect synergistic effect between ZIF-derived porous CPs and BCN nanosheets, leading to excellent capacitance of 1263 Fg^−1^ at 1Ag^−1^ and stability up to 5000 cycles. In addition, the aqueous asymmetric supercapacitors (ASCs) were also designed with redox-active Co(OH)_2_@CP@BCN as positive electrode and BCN as negative electrode which deliver high energy density of 15.55 Whkg^−1^ at 12 Ag^−1^ scan rate and stability up to 6000 cycles. We believe that presented methodology will pave new ways to tailor electrode materials from ZIF precursors to address the future energy crisis.

## Results and Discussion

**Figure 1:**
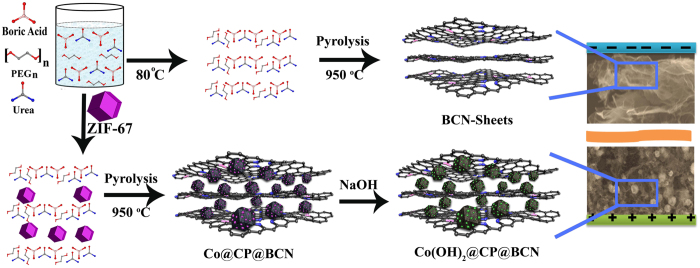
Schematic illustration for syntheses of BCN nanosheets and Co(OH)_2_@CP@BCN hybrids for asymmetric supercapacitor.

**Figure 2:**
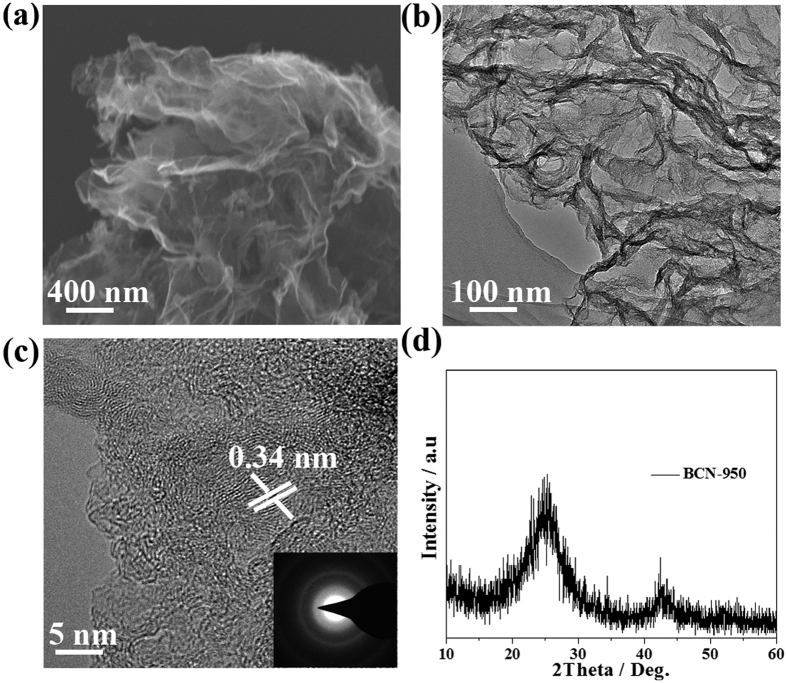
View of morphology of BCN-950 nanosheets of (**a**) FESEM, (**b**) TEM, and (**c**) HRTEM with inset image of SAED. (**d**) PXRD pattern of BCN-950.

The target BCN nanosheets were synthesized through the pyrolysis of BCN precursors at 950 °C as illustrated in Fig. 1. The morphology of BCN nanosheets was analyzed by using field emission scanning electron microscopy (FESEM) and transmission electron microscopy (TEM). The FESEM and TEM images of BCN-950 clearly show the thin highly crumpled nanosheets (Fig. 2a,b). The high resolution TEM (HRTEM) image of BCN-950 nanosheets present the amorphous structure with few graphitic lines because of the hetero atoms which shows the line spacing of 0.34 nm (Fig. 1c). The thickness of BCN-950 nanosheets was estimated by using atomic force microscopy (AFM). The thickness of BCN-950 nanosheets was not homogenous over the area of 400 nm^2^ as shown in Figure S1 (see Supporting Information (SI)). The height profile of the AFM image of BCN-950 exhibited the average thickness of sheets was about 36 nm (Figure S1b, SI). The inset selected area electron diffraction (SAED) image of nanosheets with the concentric circles clearly demonstrate the poor crystalline nature of the product, which was further confirmed by powder X-ray diffraction (PXRD) pattern with two broad peaks at 2θ values of 26 and 43° corresponding to the (002) and (100) reflection of graphitic BCN, respectively (Fig. 2d)^38^. Moreover, the overwhelming presence of B-C and N moieties (both graphitic and pyridinic) clearly suggests the successful formation of the BCN structure (Figure S2, see SI). The lower calcination temperature (850 and 750 °C) resulted in non-crumpled BCN sheets as shown in FESEM images of BCN-850 and BCN-750 (Fig. S3 and 4, see SI), implying the reaction temperature act has the vital role in making high-quality BCN nanosheets.

**Figure 3:**
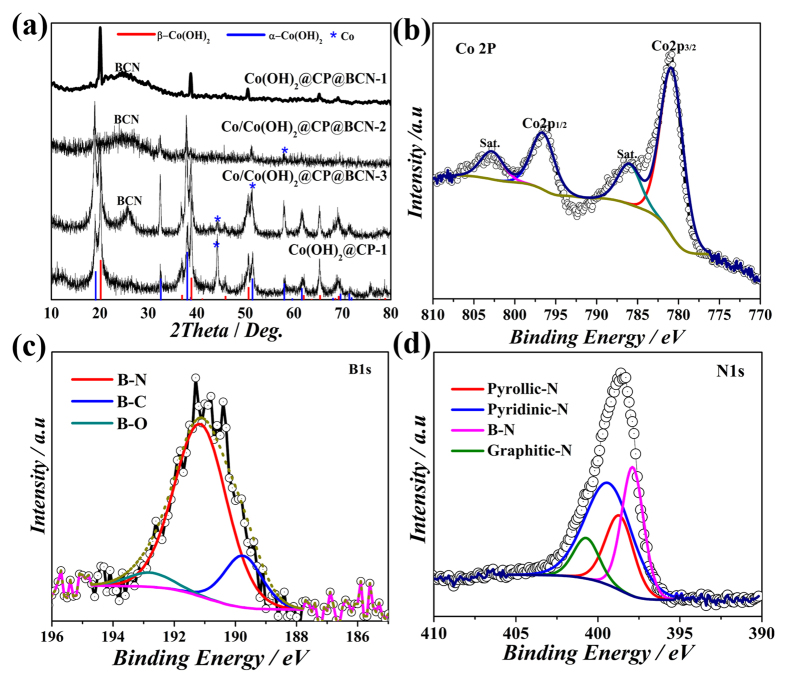
(**a**) The PXRD patterns Co(OH)_2_@CP@BCN-1, Co/Co(OH)_2_@CP@BCN-2, Co/Co(OH)_2_@CP@BCN-3, and Co(OH)_2_@CP-1 hybrids. XPS of Co 2p (**b**) B1s (**c**), and N1s (**d**) in Co(OH)_2_@CP@BCN-1.

Given the requirements of active materials for capacitive applications, Co@CP@BCN-1 hybrids were firstly fabricated by calcination of mixture of ZIF-67 nanocrystals and slurry of urea, boric acid and PEG at 950 °C. The PXRD patterns clearly confirm that Co was in metallic phase as shown in Fig. S5 (see SI). With increasing the ZIF-67 contents, higher concentrations of Co was observed in Co@CP@BCN-2 and Co@CP@BCN-3, as reflected in PXRD patterns with sharper and stronger Co peak intensity in respective products. Furthermore, these Co@CP@BCN hybrids were treated hydrothermally in 6 M NaOH solution to produce redox active Co(OH)_2_. Notedly, only Co@CP@BCN-1 could be completely converted to redox active Co(OH)_2_@CP@BCN-1 nanohybrid as confirmed by the PXRD patterns, which are well-matched with *β*-Co(OH)_2_ (card No. JCPDS-74-1057) (Fig. 3a). However, with increasing the Co source, metallic Co species appear in the hybrid as witnessed by the additional peaks appear in PXRD patterns of Co/Co(OH)_2_@CP@BCN-2 and Co/Co(OH)_2_@CP@BCN-3 hybrids (Fig. 3a). In contrast, *β*-Co(OH)_2_ and *α*-Co(OH)_2_ present in Co(OH)_2_@CP-1 (JCPDS-74-1057,73-1213 (Fig. 3a) hybrid obtained from Co@CP-1 under similar hydrothermal condition. Furthermore it was confirmed that the BCN nanosheets provide better nucleation sites for the growth of pure *β*-Co(OH)_2_ NPs. Raman spectroscopy of Co(OH)_2_@CP@BCN-1 was carried out in frequency range of 500 to 2500 cm^−1^. The D and G-bands were observed at 1353 and 1594 cm^−1^, respectively. The ratio of the intensity at D and G-bands is 1.05 which represent the graphitic structure and defects in BCN nanosheets (Figure S6, see SI).

The elemental composition of Co(OH)_2_@CP@BCN-1 nanohybrid was confirmed with 17.0% Co, 10.24% B estimated by inductive coupled plasma (ICP) and 45.27% C, 11.29% N, and 5.74% H confirmed by elemental analyzer (EA) (Table S1, see SI). The bonding configuration of these elements in Co(OH)_2_@CP@BCN-1, Co/Co(OH)_2_@CP@BCN-2 and Co/Co(OH)_2_@CP@BCN-3 nanohybrids was determined by using X-ray photoelectron spectroscopy (XPS), which show the existence of core levels of Co, B, N, C and O assures the high purity of hybrids (Figure S7, see SI). The Co2p spectra clearly depicts the two prominent shakeup satellites peaks (shown as sat.) with representative peaks at 796.9 and 780.9 eV corresponding to Co 2p_1/2_ and Co 2p_3/2_, respectively which were assigned to the signals of Co^2+^ (Fig. 3b)^39^. However, the Co/Co(OH)_2_@BCN-2 and Co/Co(OH)_2_@BCN-3 hybrids showed the metallic cobalt species at 778 eV along with Co^2+^ signals (Figure S8, see SI). Similarly, the B1s spectra can be deconvoluted into three bands which correspond to B-O, B-N and B-C, at 192.6, 191.2 and 189.6 eV, respectively^33^,^38^,^40^. The chemical state of B in hybrid confirmed its role in pining the NPs to BCN sheets for better electrochemical performance by preventing the structural detrition. Notedly, the B-C bond peaks in B1s spectra of Co/Co(OH)_2_@CP@BCN-2 and Co/Co(OH)_2_@CP@BCN-3 hybrids exhibited low intensity as compared to Co(OH)_2_@CP@BCN-1 from smaller amount of ZIF-67 precursor due to extra carbon source from 2-MeIM ligand (Figure S9, see SI). Similarly, the N1s spectra was also deconvoluted, and four different chemical states were found as B-N, pyrrolic C-N, pyridinic C-N and graphitic C-N correspond to 397.6, 398.6, 399.3 and 400.6 eV in Co(OH)_2_@CP@BCN-1, respectively (Fig. 3d). The pyridinic C-N peaks are very low in the N1s spectra of Co/Co(OH)_2_@CP@BCN-2 and Co/Co(OH)_2_@CP@BCN-3 hybrids because of the excess of ZIF-67 emulsion (Figure S9, see SI). It is well known fact that graphitic C-N and B-C species enhance the conductivity of BCN hybrid^40^,^41^,^42^,^43^. Furthermore, the contents of pyridinic (N-C) species in N-doped polyhedrons also increases at temperature above 800 °C, that enhance the electrochemical performance of the hybrid because of electroactive nature of pyridinic-N centers in carbon. The C1s spectra of Co(OH)_2_@CP@BCN-1 nanohybrid also showed three deconvoluted peaks at 286.5, 284.8 and 283.7, which correspond to the C-N, C-B and C-C bonds, respectively (Figure S10, see SI).

**Figure 4:**
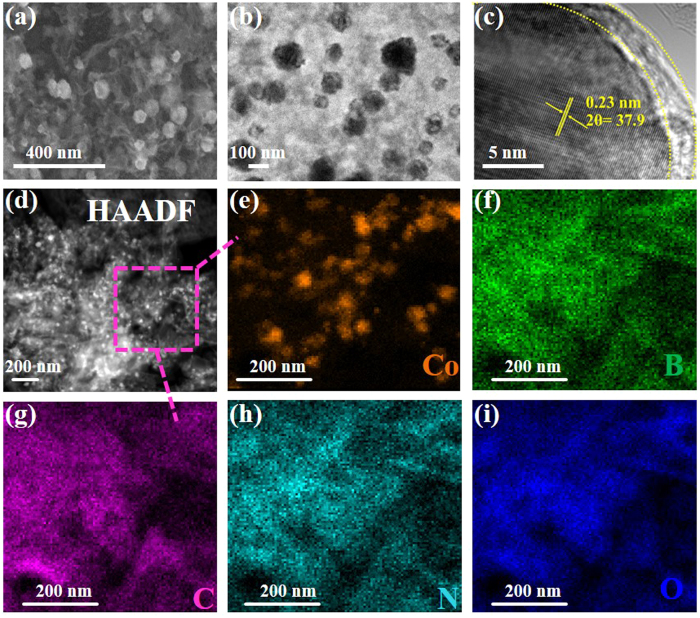
View morphology of Co(OH)_2_@CP@BCN-1 hybrid: (**a**) FESEM image, (**b**) TEM image, (**c**) HRTEM image of carbon polyhedron, (**d**) STEM image with distribution of Co (**e**), B (**f**), C (**g**), N (**h**) and O (**i**), respectively.

The ZIF-67 nanocrystals with an average size of *ca.* 300 nm were successfully fabricated, which retain their morphology in ZIF-67 derived Co@CP-1 hybrid as shown in FESEM and TEM images, respectively (Figure S11 and 12, see SI). It is well understood that pores of carbon framework could widen when subjected to strong alkaline conditions due to dissolution of surface carbon^44^. The post calcination hydrothermal treatment further reduced the polyhedra size down to *ca.* 100 nm as shown in TEM images of Co(OH)_2_@CP-1 (Figure S13, see SI). Furthermore, when grown with BCN precursors, the well decorated Co@CP on BCN nanosheets were obtained at particular temperature of 950 °C in comparison to products obtained at of lower temperature (Figure S14, see SI). After hydrothermal treatment, the Co(OH)_2_@CP@BCN-1 nanohybrid showed well defined CPs with an average size of *ca.* 100 nm decorated on the crumpled BCN nanosheets (Fig. 4a,b). These crumpling in the nanosheets should be as a result of *in-situ* B/N co-doping of carbon^35^. The additional covering of 2-MeIM on ZIF-67 nano crystals and insertion of B/N atoms in carbon framework during the pyrolysis can be beneficial for the close interaction among hybrid components. Owing to the beneficial effects of secondary sources during calcination, the derivatives of urea (cyanuric acid, melamine) and boric acid (boron oxides) tend to increase the porosity of CPs. The HRTEM studies were also carried out to confirm the structural features of hybrid, indicating the crystallinity of *β*-Co(OH)_2_ with lattice fringes of 0.23 nm with (001) plane (Fig. 4c). The elemental distribution was determined by the areal mapping and shows the presence of Co(OH)_2_ NPs inside the CPs and homogenous distribution of B, C, N and O elements as shown in the higher angle annular dark field-scanning TEM (HAADF-STEM) image at 200 nm scale bar in Fig. 4(d–i), respectively. Moreover, the products with higher ZIF-67 stoichiometric ratio led to large Co particles on the BCN sheets as shown in Figure S15,S16 (see SI). We speculate that the large size of Co particles inhibit the complete conversion of Co into Co(OH)_2_ in Co/Co(OH)_2_@CP@BCN-2 and Co/Co(OH)_2_@CP@BCN-3 hybrids.

**Figure 5:**
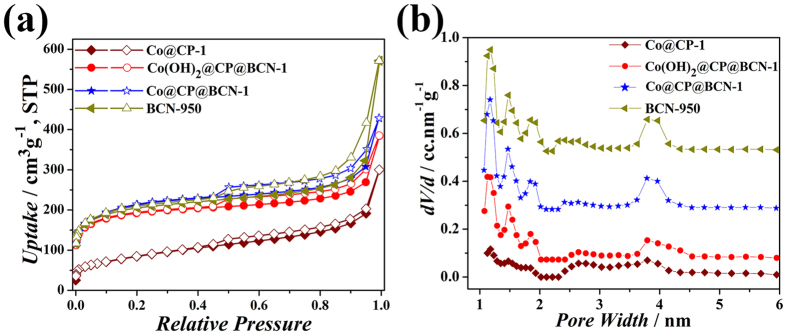
View of (**a**) N_2_ adsorption isotherms and (**b**) pore size distributions based on nonlinear density functional theory (NL-DFT) model using the adsorption points of Co@CP-1, Co@CP@BCN-1, Co(OH)_2_@CP@BCN-1 and BCN-950 at 77 K.

The electrochemical properties of electrode materials are largely determined by exposed surface and porosity. Generally, ideal electrode materials are expected to possess large surface area for enhanced electrode/electrolyte interface and hierarchical porosity for fast mass transport. The N_2_ sorption experiments were carried out at 77 K to investigate their pore volumes, Brunauer-Emmett-Teller (BET) surface area (S_BET_), and pore size distribution (PSD) based on nonlinear density functional theory (NL-DFT) model (Fig. 5a,b). The BCN-950 exhibits high S_BET_ (712 m^2^g^−1^) due to layered morphology and large amount of defects on surface. The target Co@CP@BCN-1 and Co(OH)_2_@CP@BCN-1 hybrids have been showed much higher surface area of 630 m^2^g^−1^ and 581 m^2^g^−1^ respectively, in comparison to Co@CP-1 (264 m^2^g^−1^), which indicates synergistic effect of BCN/CP in the product. The resultant Co(OH)_2_@CP@BCN-1 contained four types of interconnected pores from micropores to mesopores (1.2, 1.6, 1.8 and 3.8 nm) as shown in Fig. 5b, which is much easy to transport fully hydrated K^+^ ions (size of hydrated K^+^ 0.36 to 0.42 nm)^45^. And therefore, these pores in Co(OH)_2_@CP@BCN-1 should be able to retain the performance even at very high discharge rate without compromising over the charge storage capacity.

**Figure 6:**
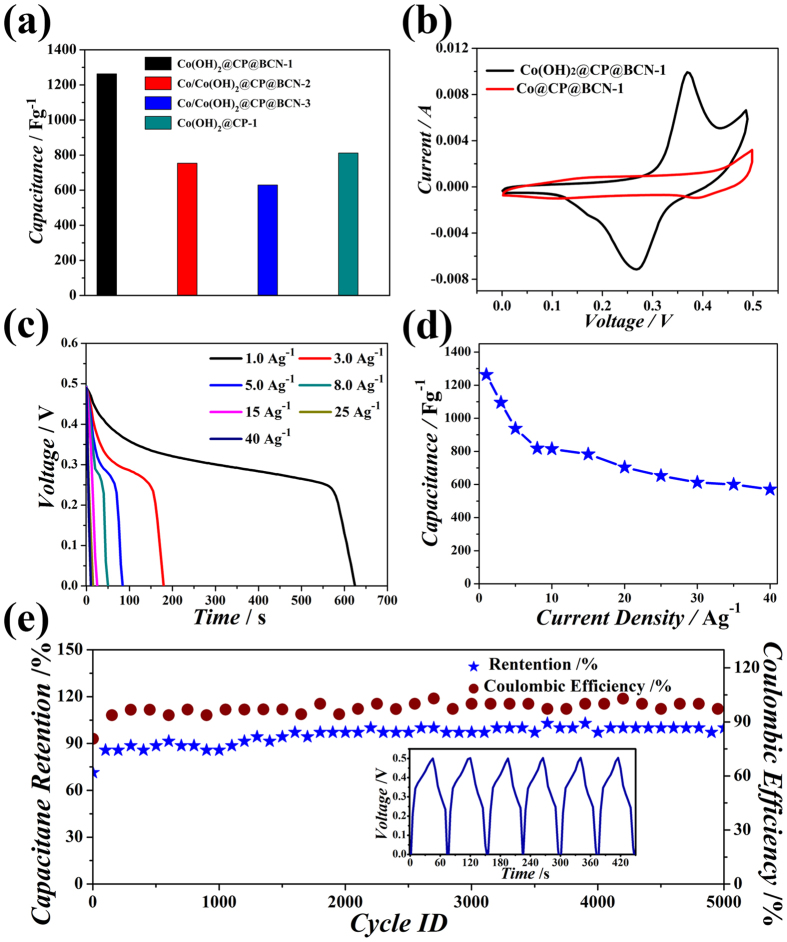
Electrochemical supercapacitor measurements in 3 electrode system (**a**) Capacitance comparison of developed hybrids. (**b**) CV curves of Co(OH)_2_@CP@BCN-1 and Co@CP@BCN-1 hybrids. (**c**) Galvanostatic discharge curves at different current density. (**d**) Capacitance values at different current densities. (**e**) Cyclic stability of Co(OH)_2_@CP@BCN-1 at current density of 10.5 Ag^−1^ up to 5000 cycles.

Considering, the high porosity, larger surface area, unique composition and two dimensional morphology of the hybrid, it is believed that the developed hybrid structure will be very beneficial for electrochemical devices. High porosity will increase the contact between electrode and electrolyte, while larger surface area will make possible maximum access to redox sites. The electrochemical activity of the hybrid will be further increased by the doping of heteroatom which can increase electro-activity of carbon by disturbing the density of state (DOS) as well as by creating partial positive and negative charges due to electronegativity difference that allows faster electronic flow and create additional redox active sites. Keeping in mind all these advantages, we examined capacitive properties of all developed hybrids. The electrochemical properties of the hybrid were tested using 3-electrode system in 6 M KOH electrolyte in 0 to 0.49 V range. The Co(OH)_2_@CP@BCN-1 hybrid has showed high specific capacitance up to 1263 Fg^−1^ among all hybrids (Fig. 6a), while the capacitances of Co/Co(OH)_2_@CP@BCN-2, Co/Co(OH)_2_@CP@BCN-3 and Co(OH)_2_@CP-1 hybrids are 758, 631 and 815 Fg^−1^, respectively. Such a high performance has been attributed due to the synergistic effect of Faradaic redox active Co(OH)_2_ nanoparticles in CPs on the BCN nanosheets. It is well understood that electrochemical activity and porosity of the developed product severely affect the charge transport. The developed hybrid takes benefit from highly porous polyhedra (contained N-doped carbon) decorated with Co(OH)_2_ nanoparticles on the B/N co doped graphene nanosheets. The N-doped carbon is deemed to be highly conductive in nature due to extra electron from the bonded N, which improve conductivity of the polyhedra while the inherent porosity (pores size 1~5.2 nm) provides pathways for quick transport of ions through 3D structure and brings better wettability of electrode to store maximum energy. The presence of B atom in hybrid also play an important role to enhance kinetics for ion transport and charge storage on electrode materials due to inherent electron deficiency in its electronic configuration can able to attract more anions in electrolyte than carbon atoms^46^. To confirm the redox active nature of developed hybrid, cyclic voltammetry (CV) of Co(OH)_2_@CP-1, Co@CP@BCN-1 and Co(OH)_2_@CP@BCN-1 hybrids was tested in the potential range from 0 to 0.5 V at a scan rate of 50 mVs^−1^ and different scan rate (Figs 6b and S17, see SI). The Co(OH)_2_@CP@BCN-1 hybrid shows clear redox peaks at 0.35 and 0.27 V, indicating the Faradaic active nature of the hybrids^33^,^47^. The possible redox reactions involve a quasi-electron transfer among Co^2+^/Co^3+^ which is facilitated by the OH^−^ ions as represented in below equations^48^,^49^,^50^.


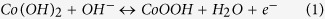



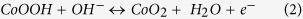


These anodic and cathodic peaks suggest excellent reversibility of redox reactions on Co(OH)_2_ in Co(OH)_2_@CP@BCN-1 hybrid. The galvanostatic charge-discharge measurements were used to further investigate the electrochemical performance. The galvanostatic discharge curves at various current densities up to very high *ca.* 40 Ag^−1^ within a potential window from 0.0 to 0.49 V (Fig. 6c). The specific capacitances values of Co(OH)_2_@CP@BCN-1 hybrid are 1263, 1095, 938, 818, 815, 783, 704, 653, 612, 600, and 571 Fg^−1^ at the current densities of 1.0, 3.0, 5.0, 8.0, 10, 15, 20, 25, 30, 35, and 40 Ag^−1^, respectively (Fig. 6d). The cyclic stability of the Co(OH)_2_@CP@BCN-1 hybrid was also examined at current density of 10.5 Ag^−1^ by consecutive 5000 charge-discharge cycles and inset of (Fig. 6e) shows its charge discharge curves. The capacity retention of Co(OH)_2_@CP@BCN-1 hybrid in first cycle was about 68% and up to 5000 cycles was about 90% capacitance retention due to the activation of electrode material, strong interactions among hybrid elements and thin carbon coating on Co(OH)_2_ nanoparticles which protect the aggregation upon cycling. Moreover, the stable electrochemical behavior of electrode was observed during the charge discharge process. The coulombic efficiency (η) defined as the ratio of discharge and charge time, is calculated from the galvanostatic experiment using the following Equation (3).


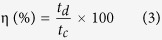


where t_c_ and t_d_ are represent the charge and discharge time, respectively.

**Figure 7:**
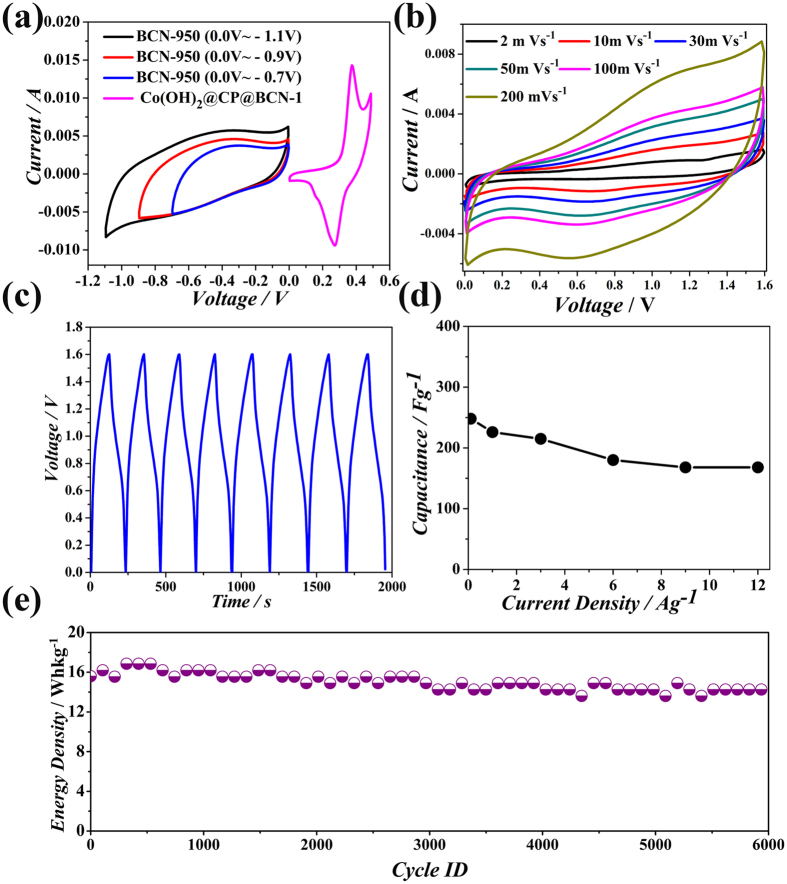
Evaluation of electrochemical performance of ASC device with Co(OH)_2_@CP@BCN-1 as positive electrode and BCN-950 as negative electrode: (**a**) CV curves for evaluating the operational window, (**b**) CV of the ASC device, (**c**) Charge discharge curves of the ASC device, (**d**) specific capacitance of the ASC device, and (**e**) stability of the ASC device.

**Figure 8:**
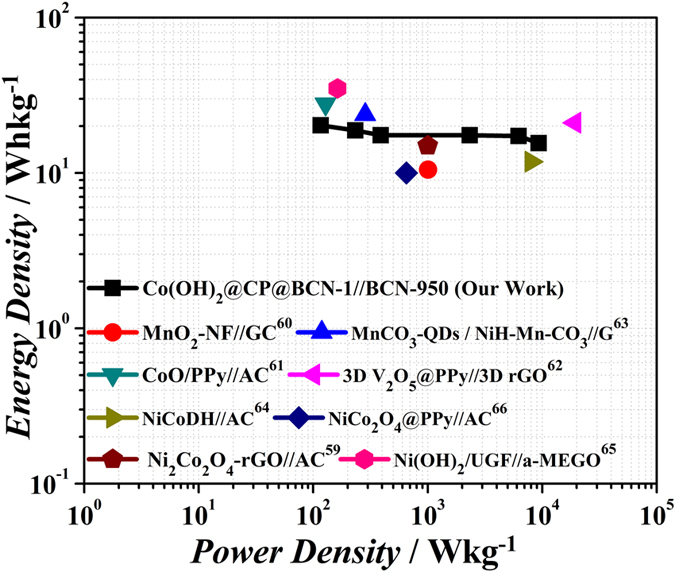
Ragone plot of ASC based on the Co(OH)_2_@CP@BCN-1//BCN-950 electrodes, showing the relationship between the energy density and power density.

To further evaluate the as-prepared Co(OH)_2_@CP@BCN-1 nanohybrid for practical application, an ASC was assembled by using the Co(OH)_2_@CP@BCN-1 as positive electrode and BCN-950 nanosheets as negative electrode. The capacitance of BCN-950 was evaluated using three electrode system in a potential window of 0.0 V to −0.7 V in 6 M KOH electrolyte (Figure S18a, see SI). Owing to unique layered morphology, binary doping and high surface area, the BCN-950 exhibited a high capacitance of 340 Fg^−1^ at current density of 1 Ag^−1^ which makes BCN-950 an ideal material for ASC as negative electrode material. Moreover, the BCN-950 nanosheets showed capacitance 340, 339, 321, 268, 265, 264, 263, 261, 254 and 251 Fg^−1^ at large charge/discharge rates of 1, 2, 3, 4, 5, 6, 7, 8, 9 and 10 Ag^−1^, indicating its suitability for ASC (Figure S18b,c, see SI). In contrast, graphene nanosheets GS-950 showed capacitance of 134 and 50 Fg^−1^ at current density of 1 and 2 Ag^−1^ (Figure S19, see SI). The water droplet angle on the BCN-850 and Co(OH)_2_@CP@BCN-1 pallets are about 72.9 ° and 55.3°, respectively which exhibited the improved wettability (Figure S20, see SI). The BCN-950 nanosheets have electrical conductivity of the 43 Sm^−1^ at room temperature because N introduces electrons and B provides holes in the system, may form the nano-junction inside the carbon framework. After deducing from above results, the higher performance of the BCN-950 is due to the high electrical conductivity, improved wettability and synergy of three atoms. In order to obtain the high performance of Co(OH)_2_@CP@BCN-1//BCN-950 device, the charge on positive and negative electrode should be optimized and based on the individual capacitive performance of Co(OH)_2_@CP@BCN-1 and BCN-950 electrodes (see SI). The ASC device can take advantage to extend the operating voltage window in aqueous electrolyte (KOH, NaOH and LiOH) up to 1.6 V^51^. For symmetric supercapacitor, applied voltage can be split because of the uniform material in each electrode. However, in ASC the maximum applied voltage depends on the charge storage capacity of individual active material^52^,^53^. Therefore, the BCN-950 electrode was measured within a stable voltage window of −1.1 to 0.0 V, while that of the Co(OH)_2_@CP@BCN-1 electrode was tested from 0.0 to 0.5 V (Fig. 7a), which is much higher than the conventional symmetric supercapacitor in aqueous electrolyte in potential range of 0.80 to 1.0 V. Accordingly, the operating potential window of 0.0–1.6 V was chosen for the overall electrochemical performances of the Co(OH)_2_@CP@BCN-1//BCN-950 ASC device for CV at different scan rates (Fig. 7b). The CV curves of the ASC device was tested in various voltage windows at a scan rate of 100 mVs^−1^ as shown in Figure S21 (see SI). The shape of CV curves looks symmetric reaching at 0.7 V and very small redox peaks appear after 0.8 to 1.1 V which can attribute to the reversible reactions and a quasi-electron transfer among Co^2+^/Co^3+^ which is facilitated by the OH^−^ ions (Figure S21, see SI). As expected, in stable operating voltage window the charge discharge curves shows the symmetric behavior as predicted in Fig. 7c at current density of 0.8 Ag^−1^. The unique morphology of Co(OH)_2_@CP@BCN-1 nanohybrid provides an easy access to a large number of ions to penetrate inside the porous sheets which gives the proper utilization of effective mass. On the other hand, BCN-950 nanosheets play an important role for providing the fast charge transfer through its compatible porosity. The ASCs exhibited high capacitance of 248 Fg^−1^ at current density of 0.1 Ag^−1^ for single electrode which is significantly higher than most of the reported ASCs. The ASCs further showed excellent rate capability with capacitance retention of 248, 226, 215, 180, 168 and 167 Fg^−1^ at current densities 0.1, 1, 3, 6, 9 and 12 Ag^−1^, respectively (Fig. 7d). In addition to higher rate of 12 Ag^−1^, the ASCs also exhibited excellent energy density of 15.55 Whkg^−1^ with over 95% capacity after 6000 cycles (Fig. 7e). The high energy density could be attributed to high capacitance of individual electrodes taking benefit from high surface area, favorable porosity, co-doping of B/N. Moreover, the presence of BCN nanosheets and pseudocapacitive material as hybrid of Co(OH)_2_@CP@BCN-1 helps to attain higher current sweeps, whereas the BCN nanosheets are mainly responsible for providing the stable and wide voltage window, which makes the contribution to high performance of ASC. The ASC device perform well in low scan rate with energy density of 20.25 Whkg^−1^ at the power density of 116.64 Wkg^−1^. In addition to higher energy density at lower scan rate, the ASC shows much high rate capability with energy density of 15.55 Whkg^−1^ at high power density of 9331 Wkg^−1^. Furthermore, the energy density and power density of ASC device was represented in Ragone plot which is the performance indicator of energy storage device^48^,^54^,^55^,^56^,^57^. A comparison of the energy density and power density obtained from our ASC device with those in previous reports is shown in Fig. 8. Even though we compare with different metal oxides and hydroxide hybrid, our device perform comparable performance to the previous reports for the energy density and power density^15^,^16^,^53^,^58^,^59^,^60^,^61^,^62^,^63^,^64^,^65^,^66^. For charge storage information, we tested electrochemical impedance spectroscopy (EIS) of the ASC device cell as shown in Figure S22 (see SI). The values of electrolyte resistance (Re) and charge (ion) transfer resistance (Rct) are 4.25 and 252 Ω, respectively. Thus the obtained performance in present work has demonstrated a novel and efficient way to improve the capacitance, energy density, power density, high rate capability, large cyclic stability of ASC using ZIF-derived CPs on BCN nanosheets. These outstanding novelties suggested the uniqueness of Co(OH)_2_@CP@BCN-1//BCN materials for their practical energy storage applications.

In summary, we have designed a strategy to decorate the ZIF-67 derived Co(OH)_2_@CPs on B and N co doped graphene nanosheets by adopting a facile method. The structural, morphological and compositional studies have shown that hybrid materials possess unique morphology that facilitate the faster ionic and electronic movements. The well-defined composition of redoxactive Co(OH)_2_ NPs into heteroatom doped carbon shell can improve the electron transport as well as boost up its electrochemical energy storage capability. The present study has proved that tuned morphology, composition and structure provide a better electrode material for energy storage systems as asymmetric supercapacitor device. The electrochemical results of present hybrid makes it potential candidate for future energy storage device.

## Experimental

### The Negative Electrode Materials

The BCN nanosheets were prepared for the negative electrode of ASC. The appropriate amount of urea (166 mmol), PEG-8000 (0.12 mmol) and boric acid (16.2 mmol) were dissolved in 100 mL water by stirring for 15 min. The as-obtained mixture was kept in oven at 80 °C for 12 h. The dried BCN precursors were annealed at 750, 850 and 950 °C in Ar atmosphere. The obtained products were recognized as BCN-750, BCN-850 and BCN-950 nanosheets.

### The Positive Electrode Materials

The Co(OH)_2_@CP@BCN-1 hybrid was fabricated for the positive electrode of ASC. In a typical experiment, a mixture of ZIF-67 crystal solution and BCN precursors was prepared, stirred for 1 h, dried at 80 °C and calcined at 950 °C for 4 h in Ar flow. To prepare the ZIF-67 solution, a stoichiometric ratio of cobalt nitrate hexahydrate (0.51 mmol) and 2-methylimidazole (2-MeIM) (22.3 mmol) were dissolved in water, mixed the BCN precursors solution was prepared as given in aforementioned procedure for BCN nanosheets and the total volume was raised to 100 mL. The reaction mixture was stirred for 30 min and kept in oven at 80 °C temperature for 10 h as shown in Scheme S1 in Supporting Information (SI). The dried form of bimixture solution was carbonized at 950 °C for 4 h in Ar flow. Similarly, the reduction of ZIF-67 polyhedra led to Co moieties on the BCN sheets termed as Co@CP@BCN-1. Since the Co is inactive for capacitive applications, the as obtained Co@CP@BCN-1 was further treated with NaOH to convert Co into Co(OH)_2_. For conversion process, 0.865 g of Co@CP@BCN-1 was dispersed in 6 M NaOH solution, sonicated for 2 h and transferred to Teflon lined autoclave for further reaction at 120 °C for 10 h. The obtained product was filtered and washed with plenty of water to remove alkaline solution (pH around 7). As a result, Co(OH)_2_@CP@BCN-1 was obtained. The products (Co/Co(OH)_2_@CP@BCN-2, Co/Co(OH)_2_@CP@BCN-3) with higher ZIF-67 concentrations were also obtained to investigate the stoichiometric effect on hybrids (Please see SI for details). Moreover, ZIF-67 derived product of Co(OH)_2_@CP-1 hybrid was also produced for comparison (see SI).

## Additional Information

**How to cite this article**: Tabassum, H. *et al*. Hierarchical Cobalt Hydroxide and B/N Co-Doped Graphene Nanohybrids Derived from Metal-organic Frameworks for High Energy Density Asymmetric Supercapacitors. *Sci. Rep.* **7**, 43084; doi: 10.1038/srep43084 (2017).

**Publisher's note:** Springer Nature remains neutral with regard to jurisdictional claims in published maps and institutional affiliations.

## Supplementary Material

Supplementary Information
