# Supplementary material for: Hierarchical Cobalt Hydroxide and B/N Co-Doped Graphene Nanohybrids Derived from Metal-Organic Frameworks for High Energy Density Asymmetric Supercapacitors
Source: Sci Rep. 2017 Feb 27;7:43084. doi: 10.1038/srep43084 (PMC5327408; doi:10.1038/srep43084)
Supplement: Supplementary Information [file srep43084-s1.doc]

Supporting Information

**Hierarchical Cobalt Hydroxide and B/N Co-doped Graphene Nanohybrids Derived from Metal-organic Frameworks for High Energy Density Asymmetric Supercapacitors**

*Hassina Tabassum, Asif Mahmood, Qingfei Wang, Wei Xia, Zibin Liang, Bin Qiu, Ruo Zhao, Ruqiang Zou**

Beijing Key Laboratory for Theory and Technology of Advanced Battery Materials, Department of Materials Science and Engineering, College of Engineering, Peking University, Beijing 100871, China. *E-mail:* [*rzou@pku.edu.cn*](mailto:rzou@pku.edu.cn)*.*

Experimental

Synthesis

**ZIF-67 Nanocrystals:** For synthesis of ZIF-67 nanocrystals[^1^](#_ENREF_1), a typical amount of cobalt nitrate hexahydrate (0.51 mmol) and 2-methyleimidazole (2-MeIM) (22.3 mmol) were dissolved in 100 mL deionized water under stirring for 30 min and left at room temperature for 10 h. After that, dispersion was washed with methanol four time by centrifugation and product was dried at 80 °C in air.

**Co@CP-1:** The ZIF-67 nanocrystals were carbonized at 950 °C with 5 °C min^-1^ heating rate for 4 h, and the resultant sample was denoted as Co@CP-1.

**Co(OH)_2_@CP-1**: 0.865 g black powder of Co@CP-1 was sonicated in 6M NaOH solution for 2 h, was transferred to teflon-lined autoclave and kept in oven at 120 °C for 10 h. After that, the mixture was filtered and washed with plenty of water to remove base solution (pH around 7). As a result, Co(OH)_2_@CP-1 was obtained.

**Co@CP@BCN-2 and Co/Co(OH)_2_@CP@BCN-2:** For synthesis of Co@CP@BCN-2 hybrid, ZIF-67 solution was prepared as aforementioned procedure only stoichiometric values of ZIF-67 precursors (cobalt nitrate hexahydrate (0.77 mmol) and 2-MeIM (33.5 mmol) were taken some high. The ZIF-67 solution and BCN precursor solution (prepared as given in synthesis of BCN nanosheets) were mixed and stirred for 1 h. After that bimixture solution was kept in oven to evaporate the solvent at 80 °C until dried completely. The dried form of bimixture solution was carbonized at 950 °C for 4 h in Ar flow. The obtained sample was named as Co@CP@BCN-2 hybrid. For the Co/Co(OH)_2_@CP@BCN-2, the 0.865 g of Co@CP@BCN-2 powder was dispersed in 6M NaOH solution and sonicated for 2 h, solution was transferred to teflon lined autoclave and kept in oven at 120 °C for 10 h. After that mixture was filtered and washed with plenty of water to remove base solution (pH around 7). As a result Co/Co(OH)_2_@CP@BCN-2 was obtained.

**Co@CP@BCN-3 and Co/Co(OH)_2_@CP@BCN-3**: For the synthesis of Co@BCN-3 hybrid, ZIF-67 solution was prepared with cobalt nitrate hexahydrate (1.54 mmol) and 2methyle imidazole (67 mmol) in 100 mL water and kept at room temperature for 12 h. The ZIF-67 solution and molecular mixed BCN precursor’s solution (prepared as given in synthesis of BCN nanosheets) were mixed and stirred for 1 h. Afterthat bimixture solution was kept in oven to evaporate the solvent at 80 °C until dried completely. The dried form of bimixture solution was carbonized at 950 °C for 4 h in Ar flow. The obtained product was named as Co@CP@BCN-3. For the Co/Co(OH)_2_@CP@BCN-3, the 0.865 g of Co@CP@BCN-3 was dispersed in 6M NaOH solution and sonicated for 2 h , solution was transferred to Teflon-lined autoclave and kept in oven at 120 °C for 10 h. Afterthat mixture was filtered and washed with plenty of water. As a result Co/Co(OH)_2_@CP@BCN-3 hybrid was obtained.

**Synthesis of graphene nanosheets:** The graphene oxide (GO) was prepared through the oxidative treatment of purified natural graphite using a modified Hummers method.[^2^](#_ENREF_2) For graphene nanosheets 100 ml GO solution was dried at 90°C. The dried GO was pyrolyzed as above annealing process and denoted by GS-950.[^3^](#_ENREF_3)

**2.3. Characterizations**

The structural analysis of the products was carried out by powder X-ray diffraction (PXRD) using Bruker D8 advanced diffractometer (Cu-Kα irradiation, 2θ = 10-70, scan rate of 4 °/min). X-ray photoelectron spectroscopy (XPS) was executed by Kratos Analytical Ltd. To determine the elemental composition analysis, a Leeman prodigy inductively coupled plasma optical emission spectrometer (ICP-OES) and Elemental Analyzer were employed. The microstructure was studied using a field emission scanning electron microscope (FESEM) and transmission electron microscope (TEM, FEI Tecnai T20) coupled with scanning transmission electron microscope (STEM). For the thickness of BCN-950 Atomic force microscopy (AFM) multimode was used. The surface area was calculated using N_2_ sorption isotherm measured in Quantachrome autosorb-IQ gas adsorption analyzer at 77 K. All samples were evacuated at 150 °C for 5 hours under dynamic vacuum before adsorption experiment. Using sorption data, nonlinear density functional theory (NL-DFT) was applied to calculate the pore size distribution while pore volumes were calculated at relative pressure (P/Po) of 0.995. For wettability, we prepared pallet of 1cm^2^ by using hydraulic presser and wettability test was performed by using the 50W quick measurement of the contact angle, the CCD camera with a resolution of 768x576pixel. The electrical conductivity of BCN-950 was measured by using cryogenic physical property measurement system (PPMS) at 300 K. The RAMAN spectroscopy of nanohybrids was carried out by using RENISHAW Raman spectrum equipment. The electrochemical measurement was carried out by using Zahner Zennium universal electrochemical workstation between potential windows of 0 to 0.49 V using Hg/HgO as reference electrode in 6 M KOH. The working electrode was prepared on nickel foam using 80% active materials. The remaining 20% of the electrode materials included binder (PTFE 10%) and active carbon (10%). For the ASC, the active materials were deposited on the Ni foam and assembled a coin type cell with 6 M KOH electrolyte, in which the BCN-950 nanosheets served as the negative electrode and Co(OH)_2_@CP@BCN-1 nanohybrids as positive electrode. The glass fiber (GF/D) from Whatman was used as a separator. The performance of ASC device was measured in the voltage window of 1 to 1.6 V **(**see SI for details).

**Electrochemical testing**

The electrochemical testing was carried out using Zahner Zennium universal electrochemical workstation between potential windows of 0 to 0.49 V using Hg/HgO as reference electrode in 6 M KOH. The working electrode was prepared on nickel foam using 80% active material. The remaining 20% of the electrode material included binder (PTFE 10%) and active carbon (10%). The mass loaded on the Ni foam was taken 1.5 to 3.5 mg.

The capacitance was calculated by using the following relationship


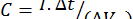


For the ASC, the active materials were deposited on the Ni foam and assembled a coin type cell of LIR 2032. The BCN-950 nanosheets were used as a negative electrode and Co(OH)_2_@CP@BCN-1 composite was used as positive electrode. Both electrodes were separated by glass fiber from the Whaatman separator and with 6M KOH electrolyte in a coin type cell. The asymmetric supercapacitor was tested in the voltage range 1 to 1.6 V. The specific capacitance of ASC was calculated by the following formula.[^4^](#_ENREF_4)


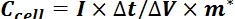


Where
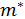
 is the total mass of two electrode. For the charge (Q) balance between both electrode (q^+^/q^-^), following relationship was used


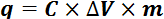


Where q is for charge, C is the specific capacitance, ΔV is the operating potential window and m is for mass. The relative mass of the positive and negative electrode was calculated by using the aforementioned relationship. For finding the mass on two electrodes the following relationship was used


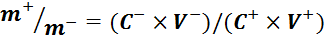


Where m^+^, C^+^ and V^+^ are for the mass, capacitance and potential window of the positive electrode and m^-^, C^-^ and V^-^ show the mass, capacitance and potential window of the negative electrode. The mass ratio of the positive and negative electrodes was measured to be ~0.66. The capacitance of single electrode in ASC was calculated by using the following relationship.[^4^](#_ENREF_4)


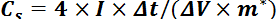


For the specific energy density of the ASC was calculated by using below relationship


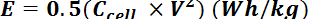


Where E is for energy density, C is for the capacitance of the ASC and V is the potential window. Moreover, for power density of the ASC following relationship was used


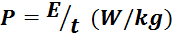


Where P represents the power density and t is for discharge time.


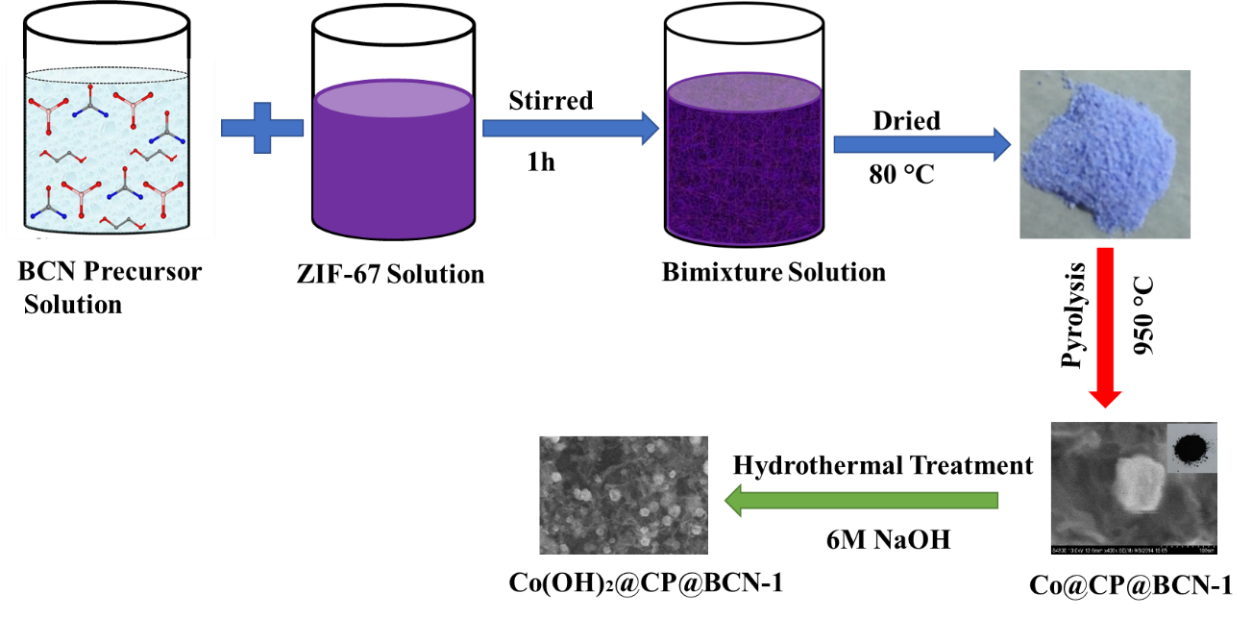


**Scheme S1**. Synthetic method of Co@CP@BCN-1 and Co(OH)_2_@CP@BCN-1.


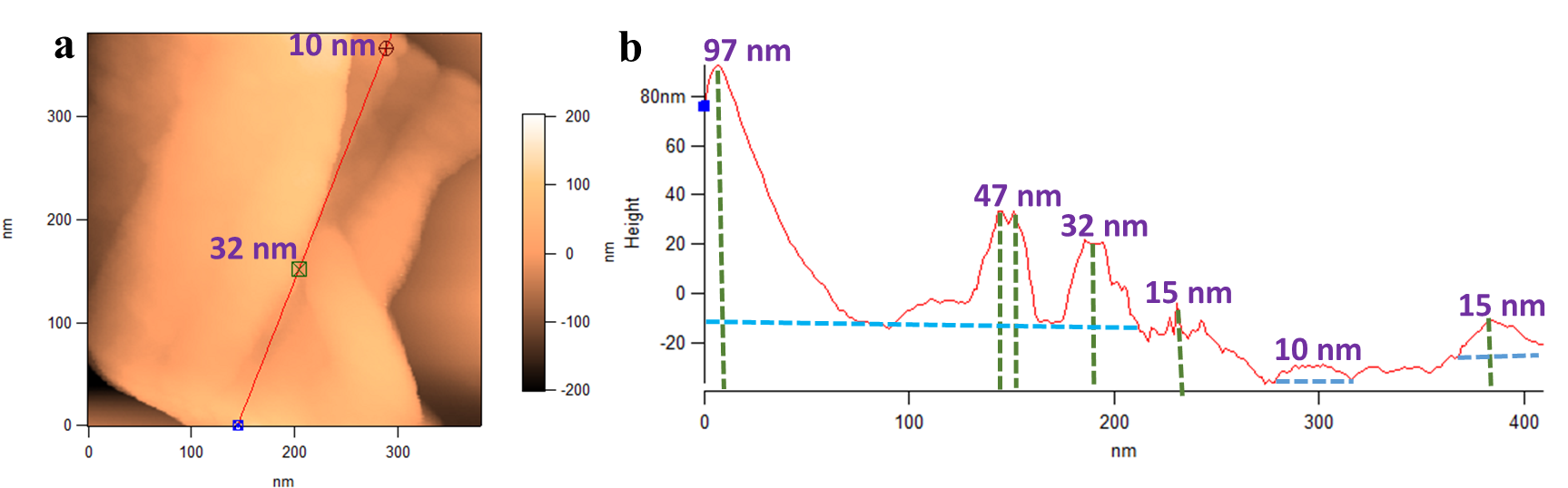


**Figure S1.** AFM (a) image and (b) height profile of the BCN nanosheets. The sheets thickness was not homogeneous. The thickness of sheets has been estimated from the height profile by drawing a line on the AFM image. As shown in height profile of AFM image of sheets, the thickness is different at different peak positions. The average sheets thickness is about 36 nm.


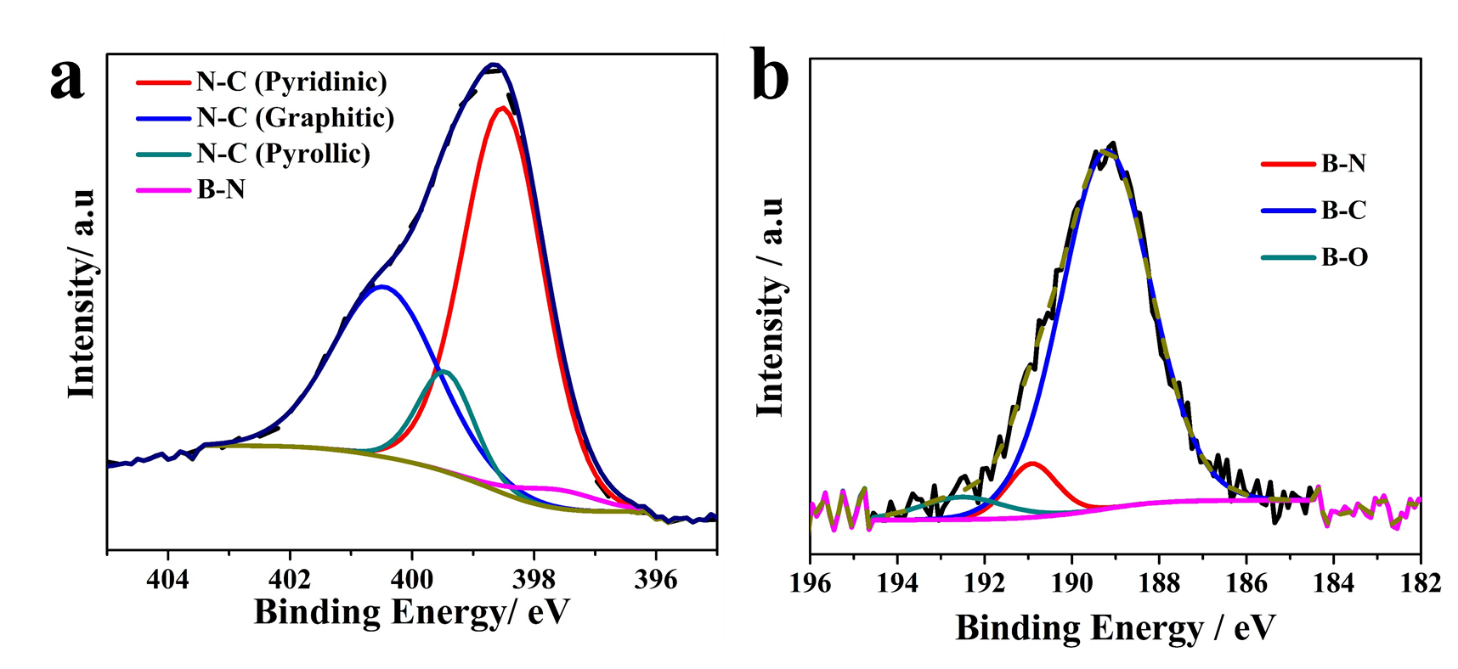


**Figure S2**. XPS spectra of (a) N1s (b) B1s in BCN-950.


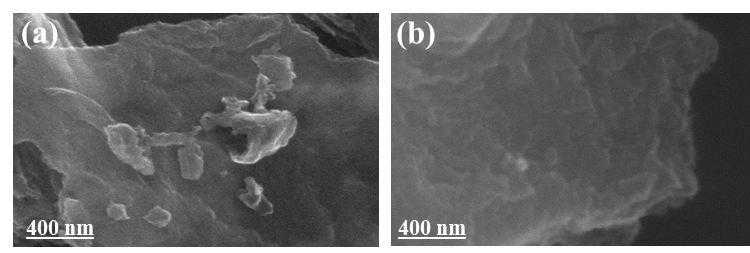


**Figure S3.** FESEM image of (a) BCN-750 and (b) BCN-850.


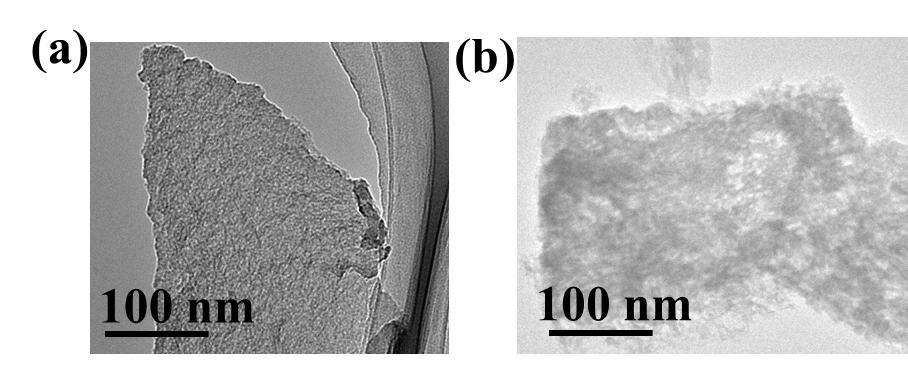


**Figure S4.** TEM image of (a) BCN-750 and (b) BCN-850.


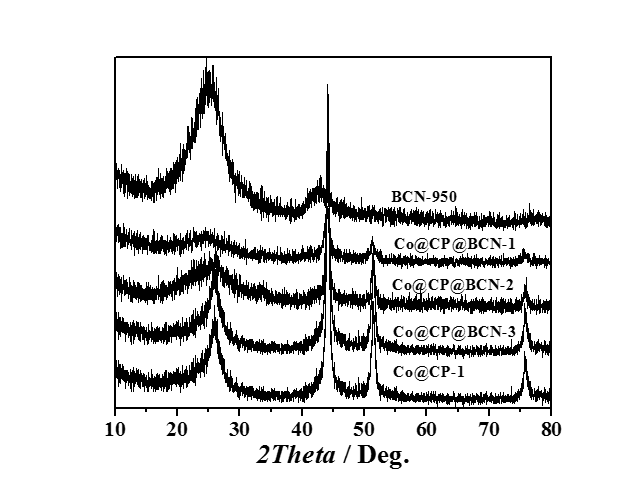


**Figure S5.** XRD pattern of Co@CP-1 and Co@CP@BCN hybrids.


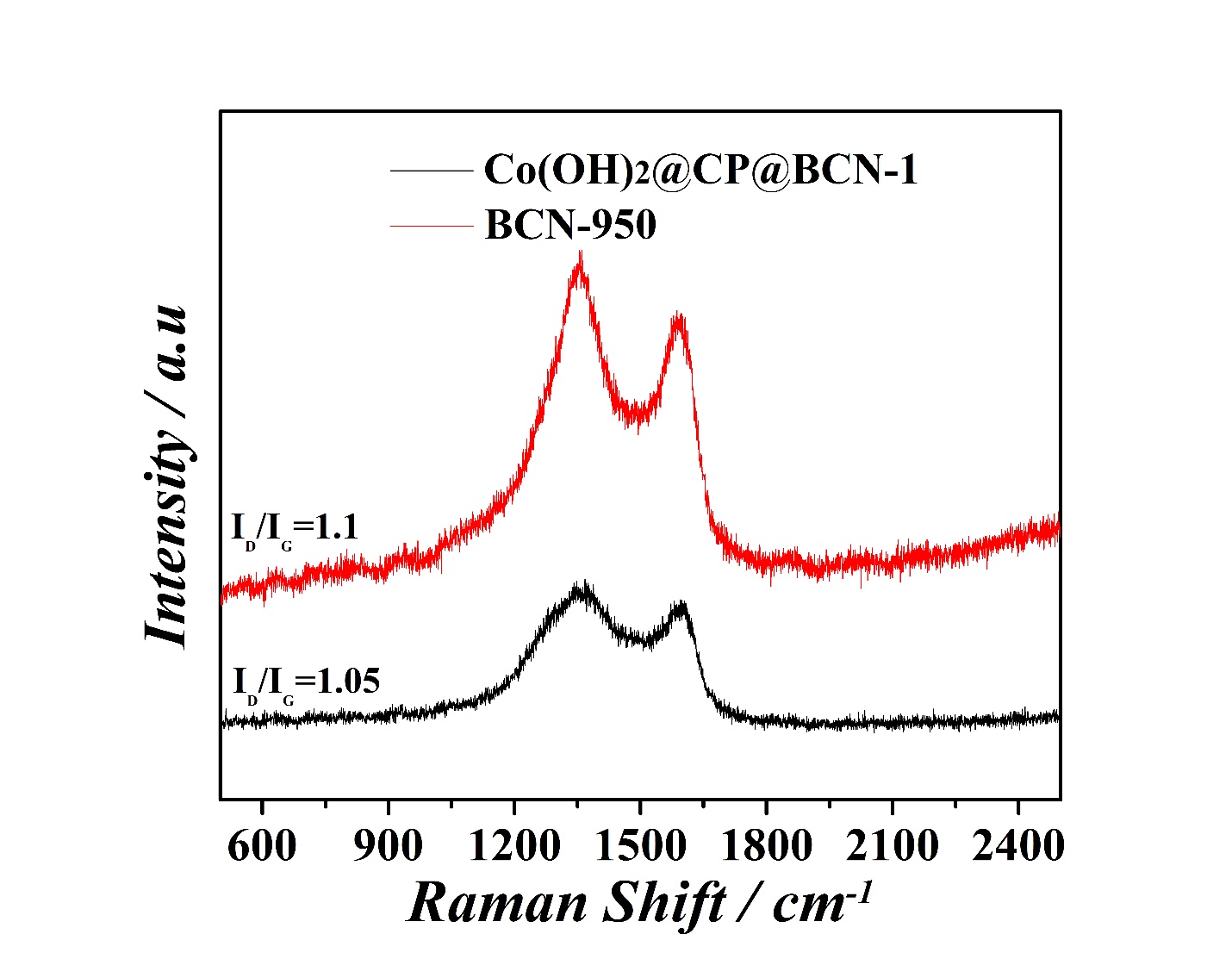


**Figure S6.** Raman spectroscopy of Co(OH)_2_@CP@BCN-1 hybrids and BCN-950 nanosheets.


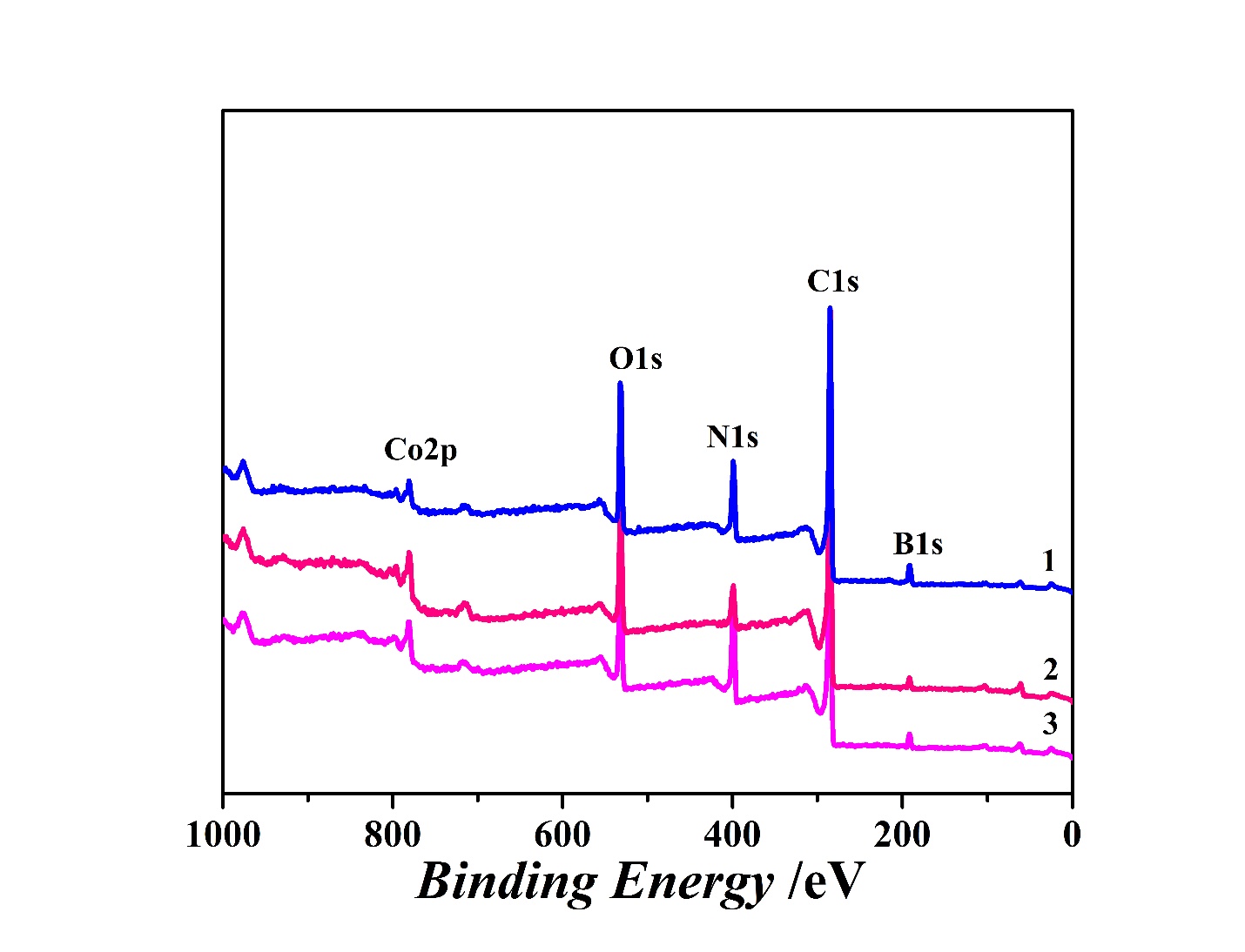


**Figure S7.** XPS spectra of Co(OH)_2_@CP@BCN-1 (1), Co/Co(OH)_2_@CP@BCN-2 (2) and Co/Co(OH)_2_@CP@BCN-3 (3) products.


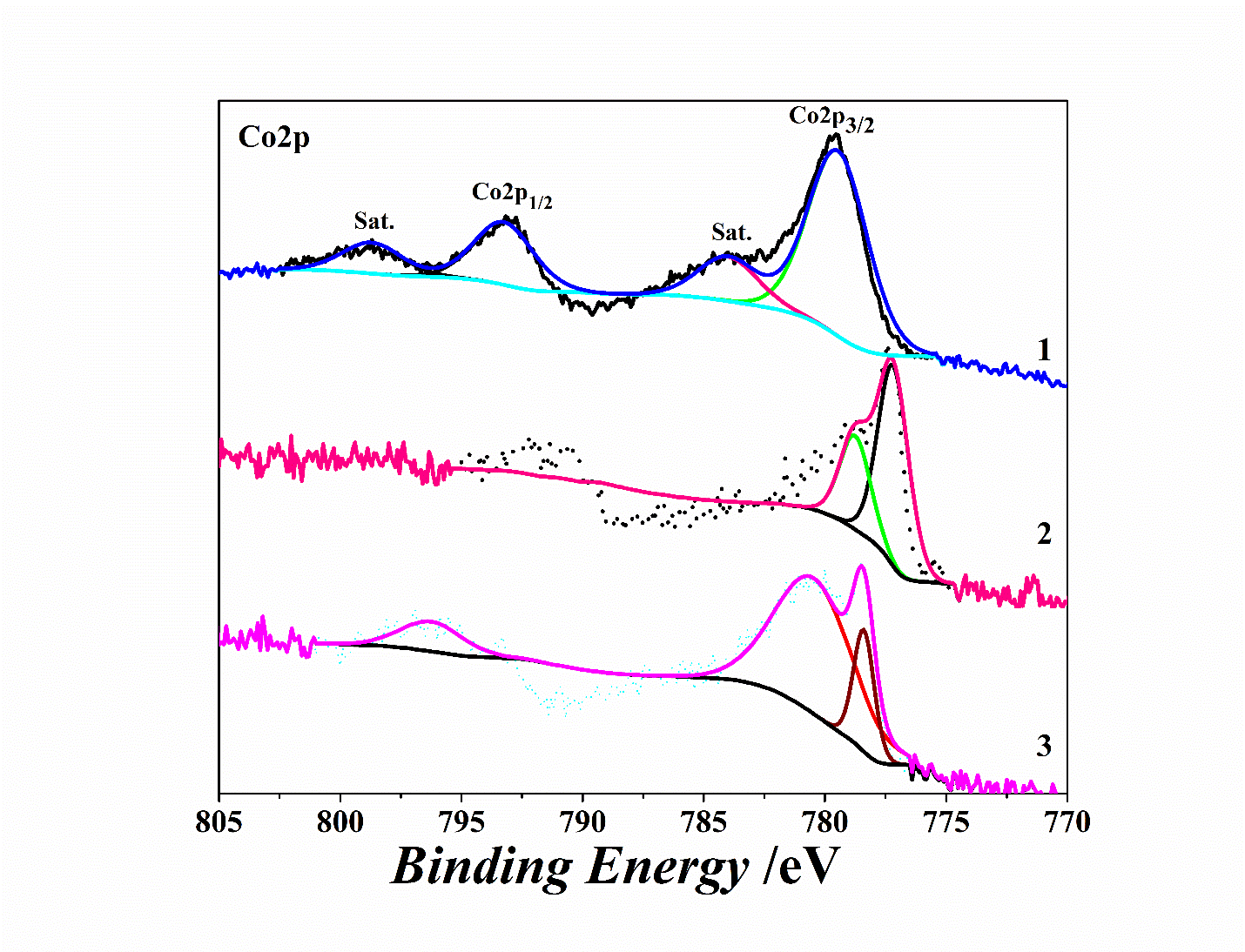


**Figure S8.** XPS spectra of Co 2p in Co(OH)_2_@CP@BCN-1 (1), Co/Co(OH)_2_@CP@BCN-2 (2), and Co/Co(OH)_2_@CP@BCN-3 (3).


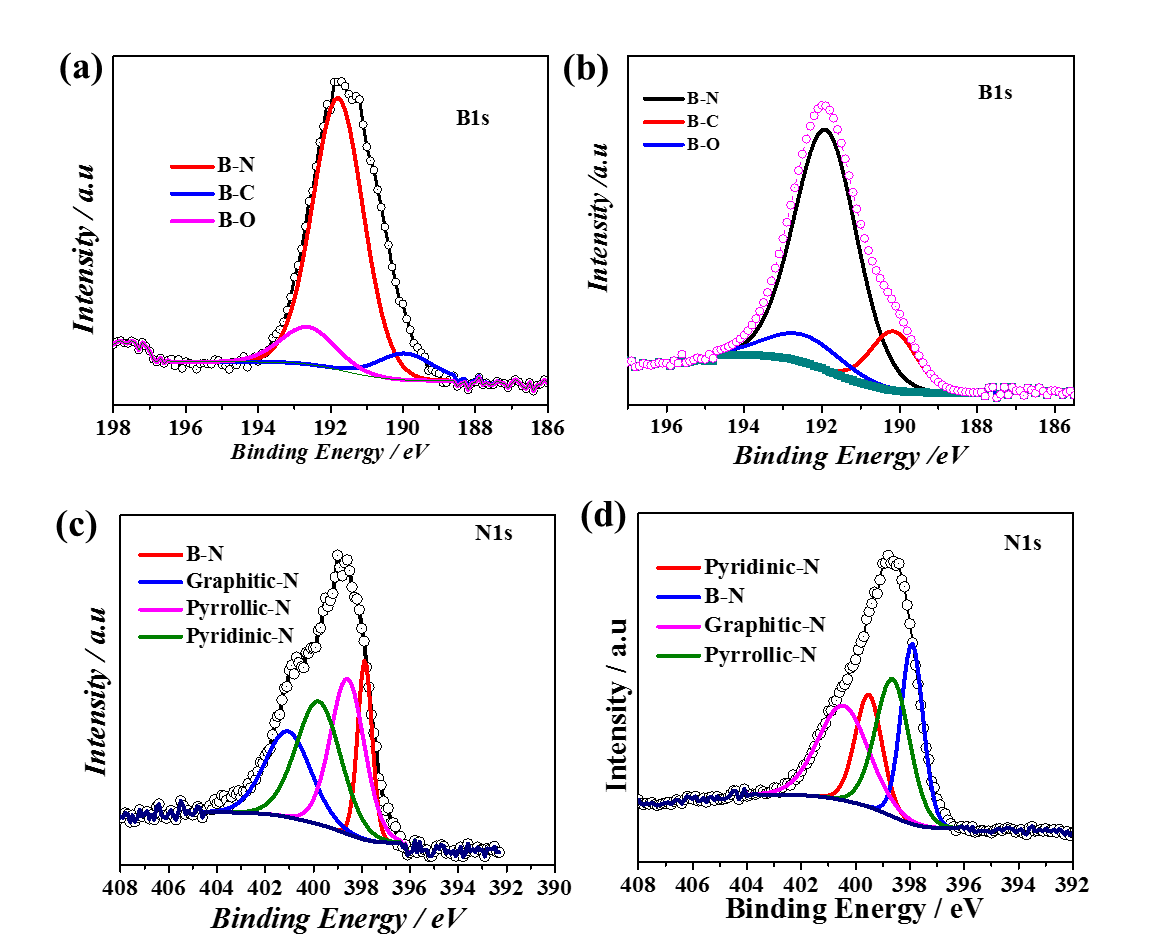


**Figure S9**. XPS spectra of (a, b) N1s and (c, d) B1s in Co/Co(OH)_2_@CP@BCN-3 and Co/Co(OH)_2_@CP@BCN-2, respectively.


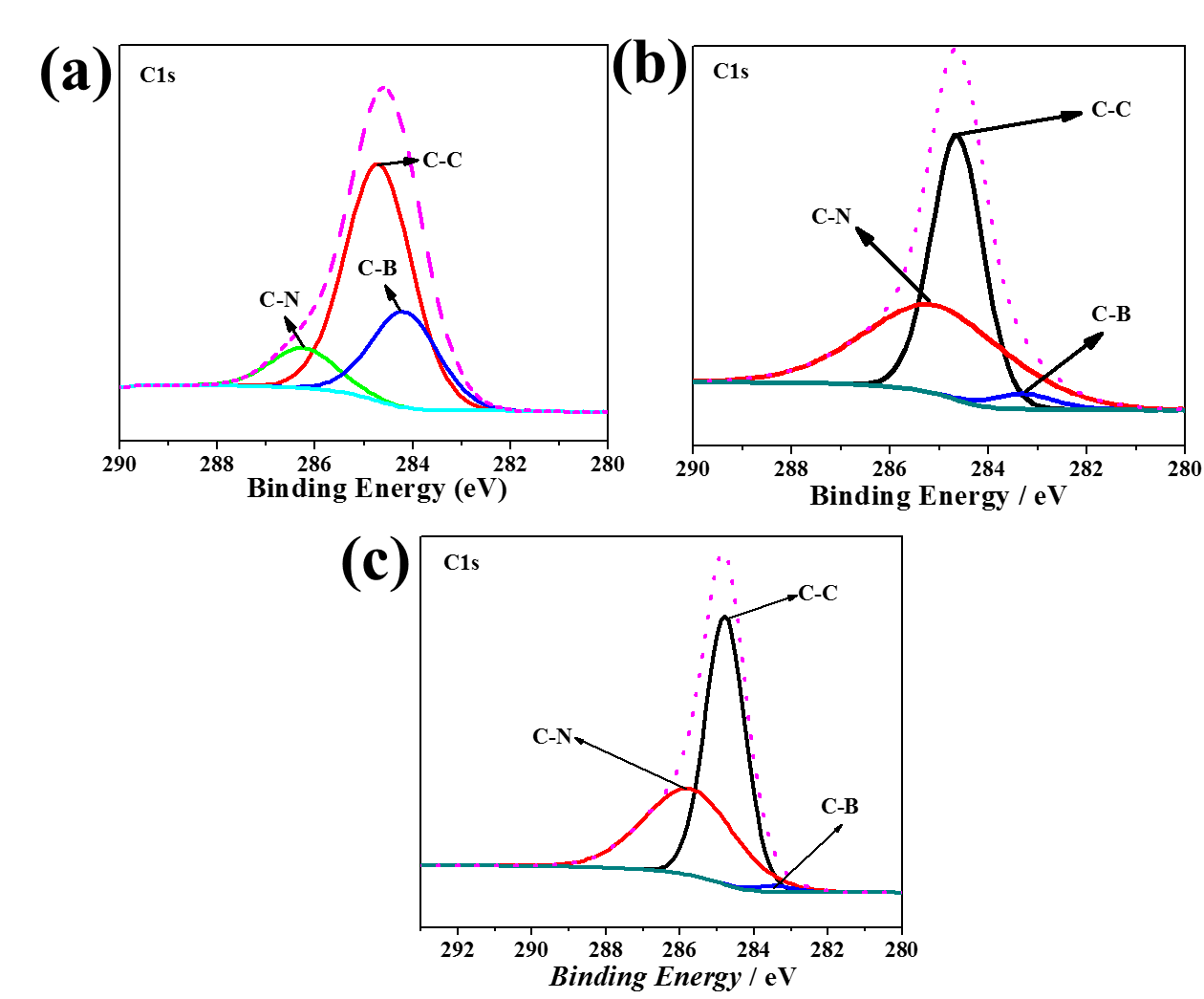


**Figure S10.** XPS spectra of C1s in (a) Co(OH)_2_@CP@BCN-1, (b) Co/Co(OH)_2_@CP@BCN-2 and (c) Co/Co(OH)_2_@CP@BCN-3.


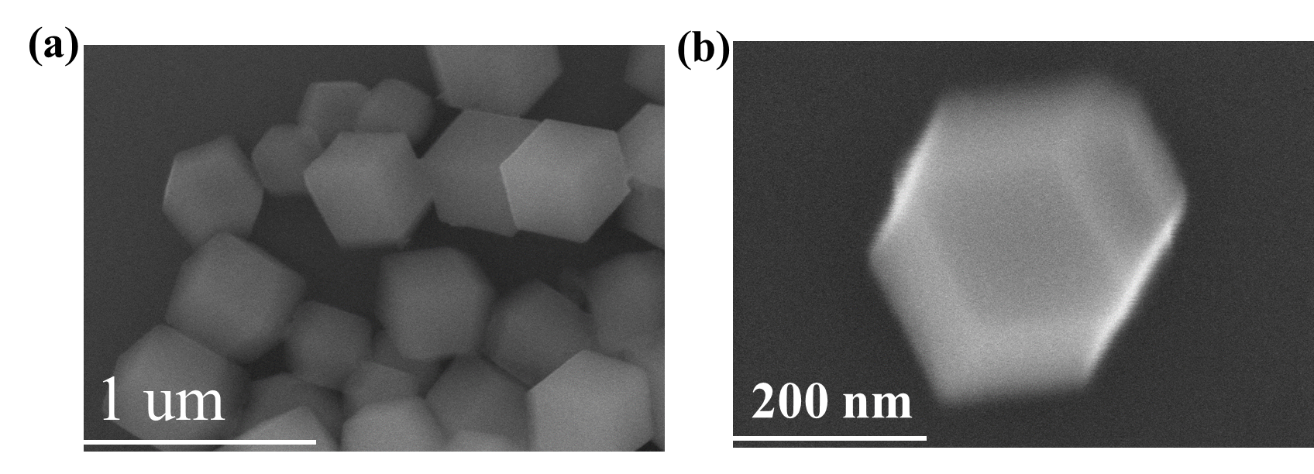


**Figure S11**. (a, b) FESEM image of ZIF-67 nanocrystals.


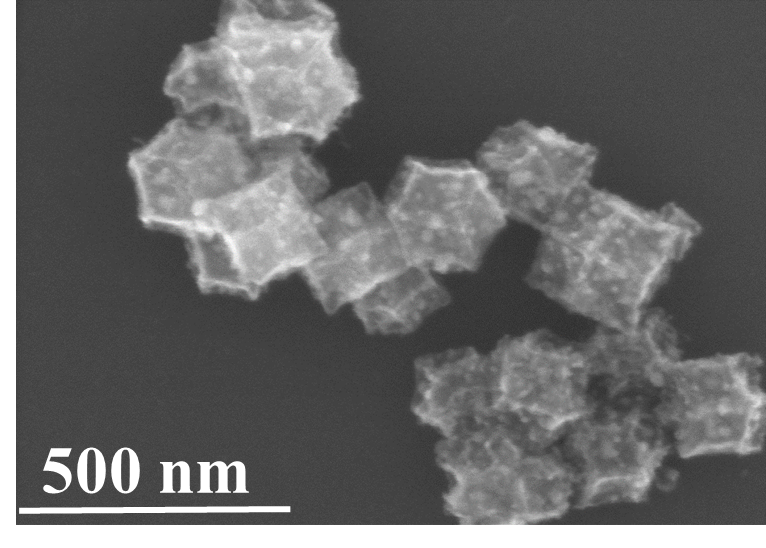


**Figure S12.** TEM image of Co@CP-1 hybrids.


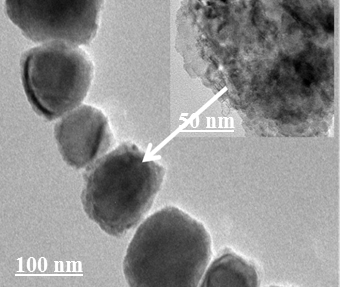


**Figure S13**. TEM image of Co(OH)_2_@CP-1 hybrids.


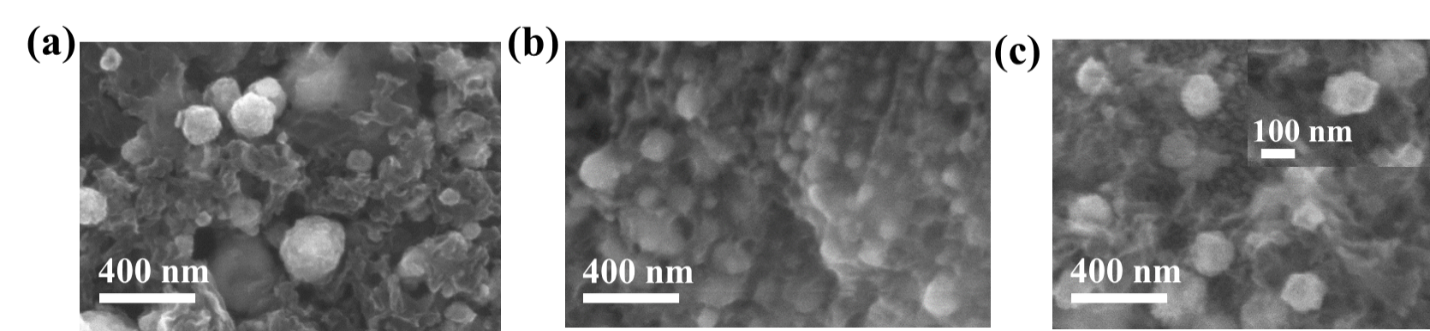


**Figure S14.** FESEM images of Co@CP@BCN-1 at (a) 750°C, (b) 850°C and 950°C.


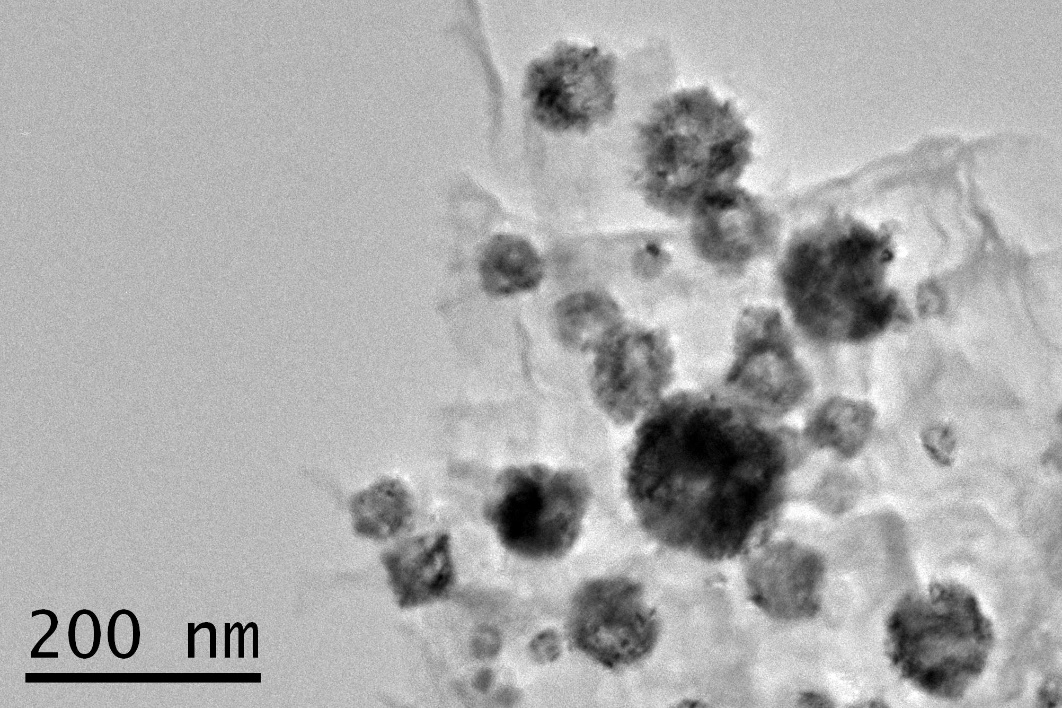


**Figure S15.** TEM image of Co/Co(OH)_2_@CP@BCN-2.


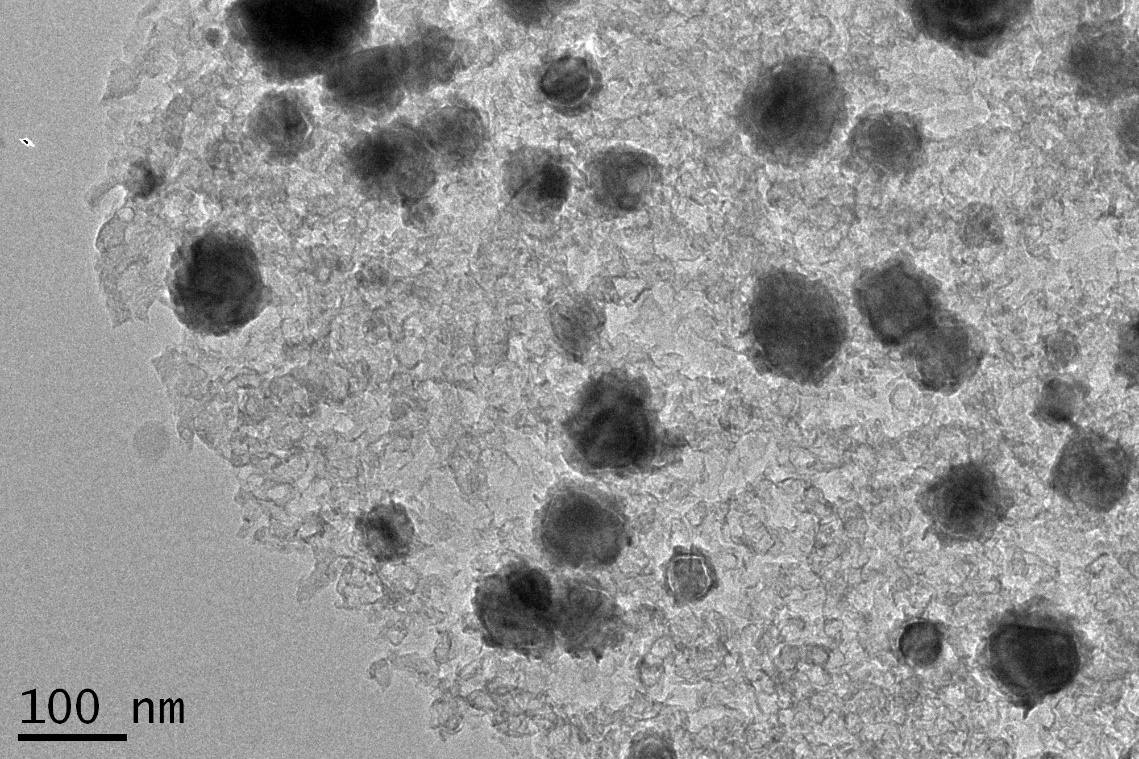


**Figure S16**. TEM image of Co/Co(OH)_2_@CP@BCN-3.

**Table S1.** Elemental Contents results from ICP (for B, Co) and EA (for C, N, H) results.

| Sample Name | Co% | B% | C% | N% | O% | H% |
| --- | --- | --- | --- | --- | --- | --- |
| Co(OH)_2_@CP@BCN-1 | 17.00 | 10.24 | 45.27 | 11.29 | 10.57 | 5.54 |
| Co/Co(OH)_2_@CP@BCN-2 | 20.00 | 10.47 | 49.75 | 11.63 | 6.12 | 2.02 |
| Co/Co(OH)_2_@CP@BCN-3 | 24.30 | 9.29 | 50.70 | 9.40 | 4.65 | 1.64 |


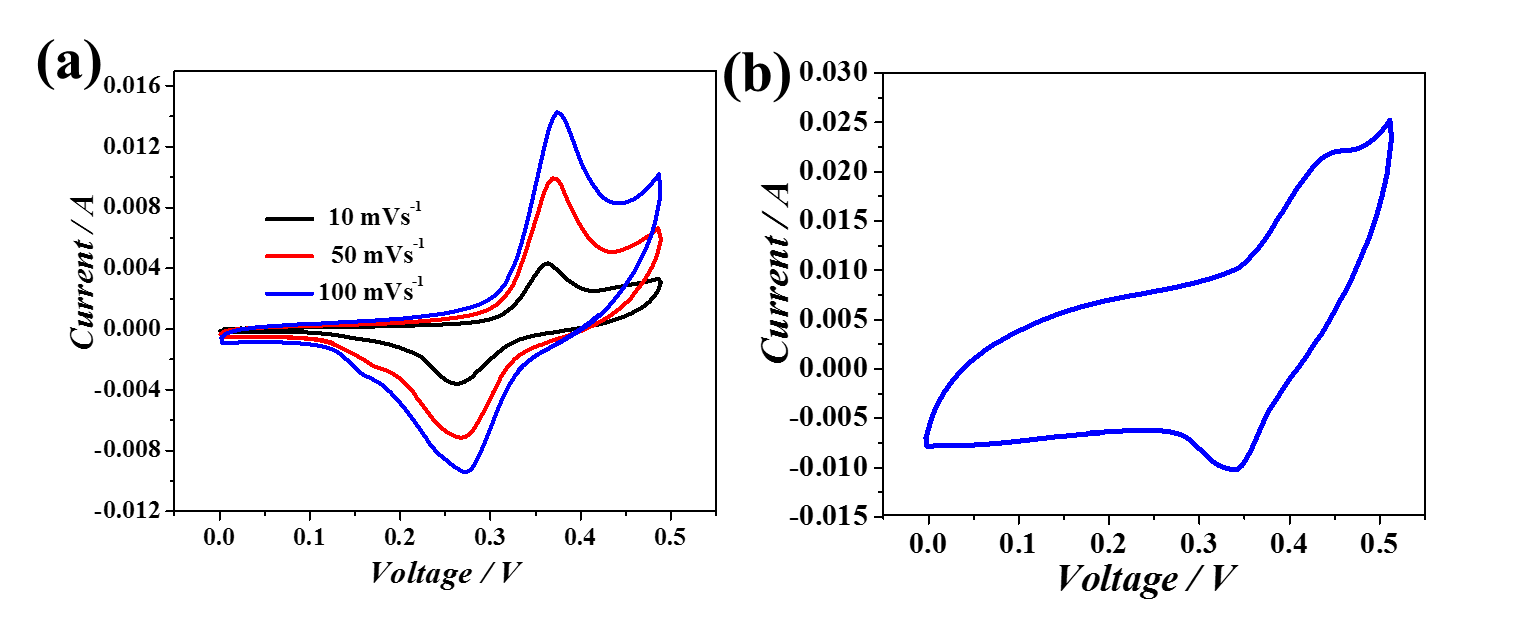


**Figure S17.** (a) CV curves of Co(OH)_2_@CP@BCN-1 at different scan rate, (b) CV of Co(OH)_2_@CP-1.


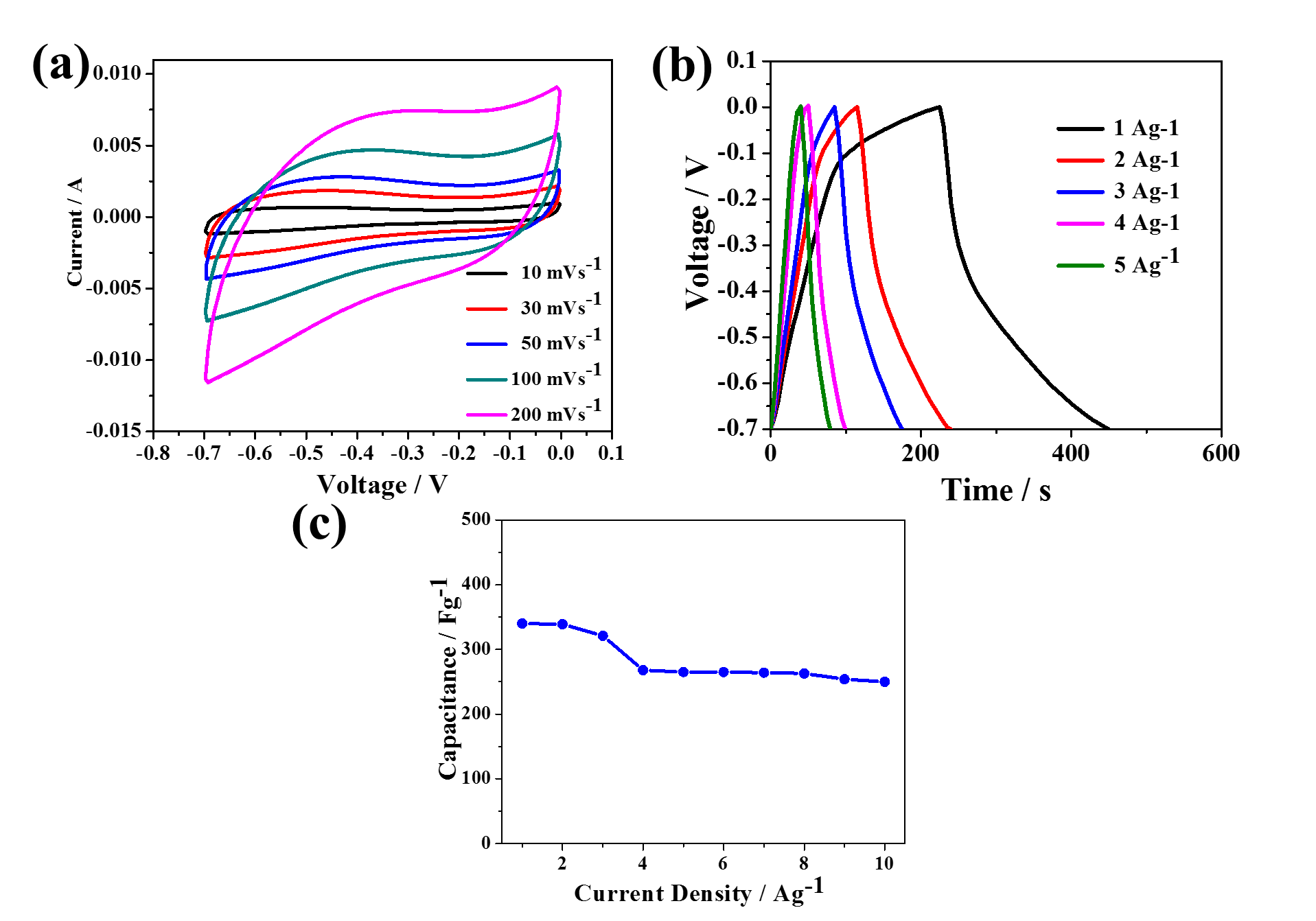


**Figure S18.** View of (a) CV, (b) discharge cycle, and (c) capacitance curves of BCN-950.


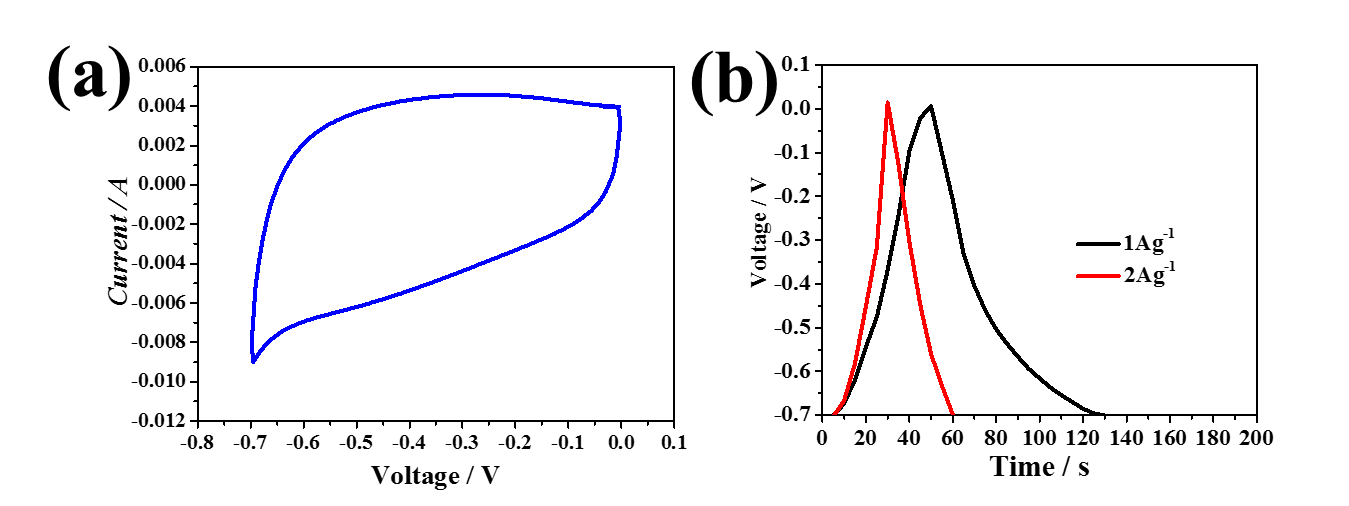
 **Figure S19**. (a) CV and (b) charge-discharge curves of graphene.


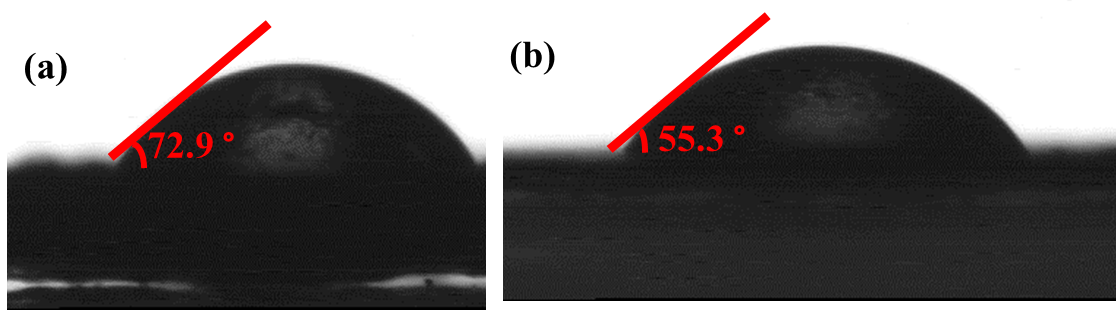


**Figure S20.** Droplet angle for Wettability test of the (a) BCN-950 and (b) Co(OH)_2_@CP@BCN-1 hybrid.


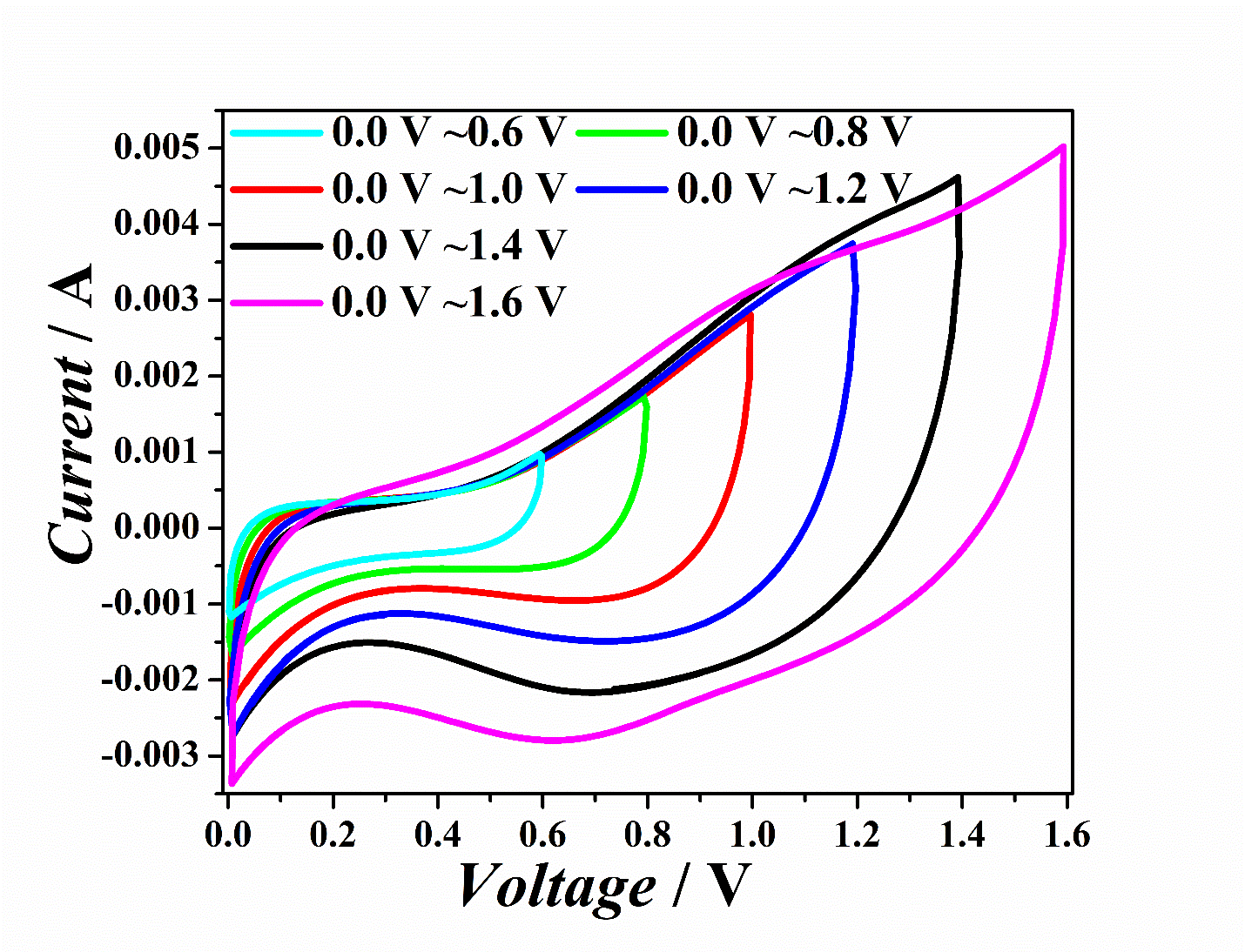


**Figure S21.** CV curves of ASC for evaluating the operational window.


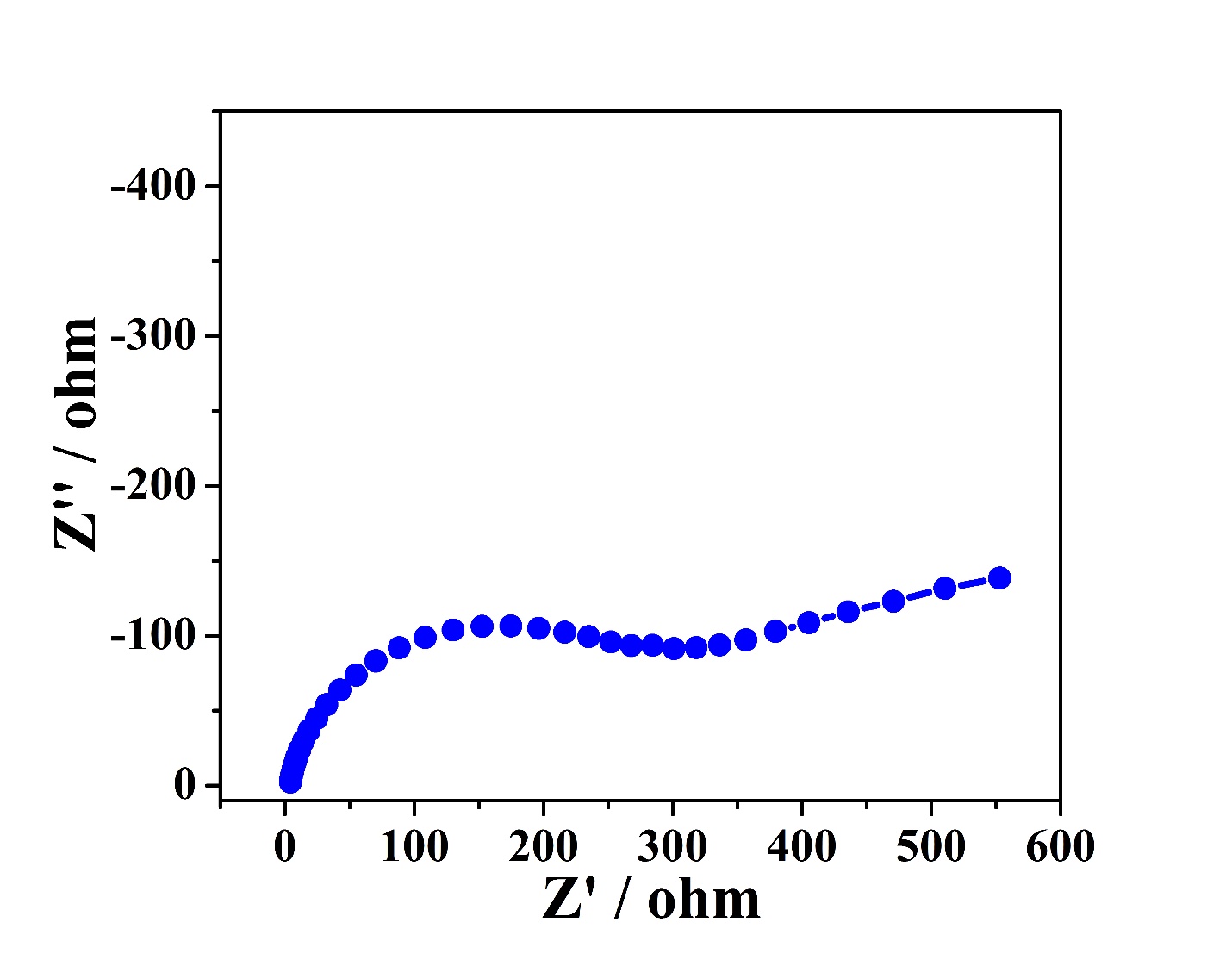


**Figure S22**. EIS of the Co(OH)_2_@CP@BCN-1//BCN-950 ASC device.

**References**

1 Xia, W. et al. Well-defined carbon polyhedrons prepared from nano metal–organic frameworks for oxygen reduction. *J. Mater. Chem. A* **2**, 11606 (2014).

2 Hummers, W. S. & Offeman, R. E. Preparation of Graphitic Oxide. *J. Am. Chem. Soc.* **80**, 1339-1339 (1958).

3 Wen, Z. et al. Crumpled Nitrogen-Doped Graphene Nanosheets with Ultrahigh Pore Volume for High-Performance Supercapacitor. *Adv. Mater.* **24**, 5610-5616 (2012).

4 Stoller, M. D. & Ruoff, R. S. Best practice methods for determining an electrode material's performance for ultracapacitors. *Energy Environ. Sci.*  **3**, 1294-1301 (2010).
